# Regulation of Pore Evolution via Progressive Electroporation Enhanced Intracellular Molecule Transport

**DOI:** 10.34133/research.1095

**Published:** 2026-01-23

**Authors:** Xiao-Nan Tao, Xiao-Wei Xiang, Hao-Tian Liu, Cai-Hui Zhu, Jing Liu, Ya-Jun Wang, Wei Liu, Yu-Chen Chen, Yu-Lian Zeng, Sai-Xi Yu, Jian Qiu, Guangyin Jing, Hui Zhao, Qihong Huang, Yan-Jun Liu, Ke-Fu Liu

**Affiliations:** ^1^ School of Information Science and Technology, Fudan University, Shanghai 200433, China.; ^2^ Academy for Engineering & Technology, Fudan University, Shanghai 200433, China.; ^3^ Shanghai Key Laboratory of Medical Epigenetics, International Co-laboratory of Medical Epigenetics and Metabolism (Ministry of Science and Technology), Institutes of Biomedical Sciences, Fudan University, Shanghai 200032, China.; ^4^ Ruijin Hospital, Shanghai Jiao Tong University School of Medicine, Shanghai 200025, China.; ^5^ School of Physics, State Key Laboratory of Photon Technology in Western China Energy, Northwest University, Xi’an 710127, China.; ^6^ Zhongshan Hospital Institute of Clinical Science, Shanghai Medical School, Fudan University, Shanghai 200032, China.

## Abstract

Nonviral intracellular delivery based on pulsed-electric-field-induced electroporation is one of the most effective and widely used platforms in basic biological and biomedical research. However, the conventional bulk electroporation technique has exhibited limited performance in improving delivery efficiency with a single type of pulse, especially for in vivo small interfering RNA (siRNA) delivery. Pulse modulation has been confirmed effective in facilitating intracellular delivery. Nonetheless, pore evolution and regulation during and after electric exposure plays an essential role in the effective intracellular delivery of molecules with variable sizes. Here, we propose a progressive electroporation (PEP) strategy on the basis of multiple-pulse combination, which decouples the perforation process and delivery process compared to conventional bulk electroporation, efficiently improving delivery efficiency with regulation of the perforated pores. We demonstrated an important correlation between delivery efficiency enhancement and delayed pore resealing by quantitative investigations. The performance of this disruption-and-field-enhancement method also showed delivery advantages over conventional chemical systems. Moreover, we validated the improvement for siRNA knockdown efficacy in vivo. Overall, PEP helps provide a unique insight into improving intracellular delivery, by regulating pore dynamics rather than just inducing perforation. This strategic advancement of PEP may pave the way for the development of advanced wearable delivery systems with reduced energy consumption.

## Introduction

Intracellular delivery, which involves introducing exogenous materials into cells, is pivotal in universal biological and biomedical research [1,2]. Gene therapy, especially small interfering RNA (siRNA)-based therapy, has gained broad attention for its great potential in both basic research and clinical applications [3]. Currently, a plethora of siRNA drugs have entered the clinical stage [4]. However, challenges in delivering siRNA such as membrane penetration inability and ineffective endosomal escape have hindered the advancement of nucleic therapeutics [5–7]. Compared to systemic administration, local administration can prevent the degradation of siRNA in the blood, whereas the ability of drugs to cross the cell barrier must be considered [3]. Therefore, robust intracellular delivery techniques are imperative to augment the utility and efficacy of siRNA-based applications [6].

Numerous techniques have been developed for penetrating the cell barrier, encompassing biological, chemical, and physical approaches [8–10]. However, biological and chemical vectors such as viruses and liposomes can be immunogenic, which may raise serious safety issues in vivo [11]. Comparatively, physical methods based on temporary membrane disruption provides feasible and simple solutions to intracellular delivery [12]. Among these methods, electroporation, which utilizes high-voltage short-duration pulses (HSPs) to overcome cell membrane barriers, has been used in both in vitro and in vivo transfection due to its versatility and applicability to a diverse variety of cells and tissues [13,14]. However, the conventional bulk electroporation technique employing a single type of pulse exhibits constrained potential to enhance delivery efficiency in in vivo siRNA applications. This limitation arises from the cytotoxicity associated with high siRNA concentrations, whereby achieving efficient intracellular delivery at reduced siRNA doses becomes imperative to balance therapeutic efficacy and biosafety [3,15].

Focused on the electroporation process, conventional electroporation implements a modest external electric field for the polarization of the membrane phospholipid bilayer [16], which triggers transient and reversible conformation of the membrane, providing channels for endogenous substances, such as nucleotides, to enter the cytoplasm through the “electropores”. The typical electric parameters for conventional bulk electroporation are 8 pulses of 100 μs, 1,300 V/cm, and 1 Hz [17]. After removing the electric field, the cell membrane undergoes a stabilizing process, enclosing the nucleotides in the cytoplasm, which renders effective transfection [18]. However, electric pulses of varying intensities serve distinct functions: high-intensity pulses primarily induce electroporation, whereas low-intensity pulses facilitate electrophoresis. As a physical-field-mediated method, the role of the pulsed electric field (PEF) dominates the efficacy of membrane electroporation and molecule transport [19]. Nonetheless, insufficient mechanistic understanding and lack of rationality for pulse modulation manner and voltage distribution further limit electroporation and delivery performance [12]. Over the decades, researchers have pursued enhancements through pulse modulation. It has been acknowledged that pulse voltage magnitude and pulse duration are 2 crucial factors that have been reported to dominate the numbers and sizes of electropores, respectively, by numerical simulation [20]. Hence, these 2 factors need to be carefully considered before electroporation [21]. The high voltage and lack of electric dose control in conventional electroporation techniques can cause severe damage to cells, even triggering cell death [22,23], which limits the widespread application of electroporation [24].

Studies involving the trade-off between viability and transfection efficacy have shown that the multiple-pulse strategy outperforms the single-pulse strategy in vivo [25]. Integration of low-voltage (LV) pulses into HV pulses has shown advantages over the conventional bulk electroporation technique where the transport of molecules with charges can be influenced by electrophoresis as reported [26,27]. The electrophoretic effect was also verified by Ding et al. [28] combining cell squeezing and electric-field-driven transport for membrane disruption and active transport of DNA to the nucleus, respectively, which exhibited rapid expression with minimal cell damage. Compared to conventional bulk electroporation, multiple-pulse-mediated membrane permeabilization and enhanced DNA transport by electrophoresis can ameliorate the transfection efficacy, reducing the proportion of HV components and maintaining cell viability [19,29]. However, previous studies have primarily focused on the effects of multiple pulses in enhancing membrane electroporation [30] or improving the transport of charged molecules [29]. To our knowledge, few investigations have addressed the regulation of post-electroporation pore expansion and resealing through pulse combinations [31,32], despite their broad applicability for diverse molecular cargos [32]. Diffusion, as well as electrophoresis, contributes to siRNA delivery on a longer time scale during pore maintenance compared with the immediate effects of the electric field [33]. Regulation of pore resealing dynamics represents a previously underexplored mechanism that is nevertheless critical for delivery efficiency, which may help constrain electric energy input and minimize cellular damage. Therefore, revisiting the effects of pulse composition on electropore evolution post-electroporation is essential for rationally optimizing pulse modulation to enhance intracellular delivery efficiency while preserving high cell viability.

In this study, we revealed that pulse modulation played a regulatory role in pore closure dynamics using the nonviral intracellular delivery strategy of progressive electroporation (PEP). HV microsecond pulses with shortened duration and total treatment time effectively induced membrane electroporation while preserving cell viability, followed by LV millisecond pulses to perturb pore expansion and resealing. By decoupling membrane electroporation and molecular delivery, pulse combination induced a different regulatory mechanism compared to a single HV component. To better understand the process, we systematically analyzed the effects of LV millisecond pulses on the expansion and resealing trends of hydrophilic pores based on established theoretical models of electroporation. By limiting the decay of the transmembrane voltage (TMV), LV millisecond pulses modulated fluctuations in pore energy, thereby delaying the minimization of pore free energy in a reversible manner. Experimentally, we observed delayed pore resealing along with enhanced delivery efficiency over a range of molecules without causing significant cytotoxicity. Moreover, we confirmed the potential of PEP in facilitating in vitro delivery of siRNAs for the suppression of target genes, which showcased comparable efficiency (57% versus 55%) to chemical lipofection while improving cell viability (70% versus 43%). We validated the in vivo applicability as well. When compared with that of conventional bulk electroporation, the electrical energy consumption of PEP rendered a reduction of 17%. Overall, this study provided a mechanistic, model-based demonstration of effective membrane electroporation induced by HV pulses and the subsequent regulation of pore evolution by LV pulses, which was further validated by experimental observations. These findings offered important insights for the improvement of intracellular delivery strategies based on electroporation by pulse modulation. With higher efficiency and lower energy consumption than conventional bulk electroporation, this strategy could accelerate the development of next-generation wearable delivery systems.

## Results

**Fig. 1.**
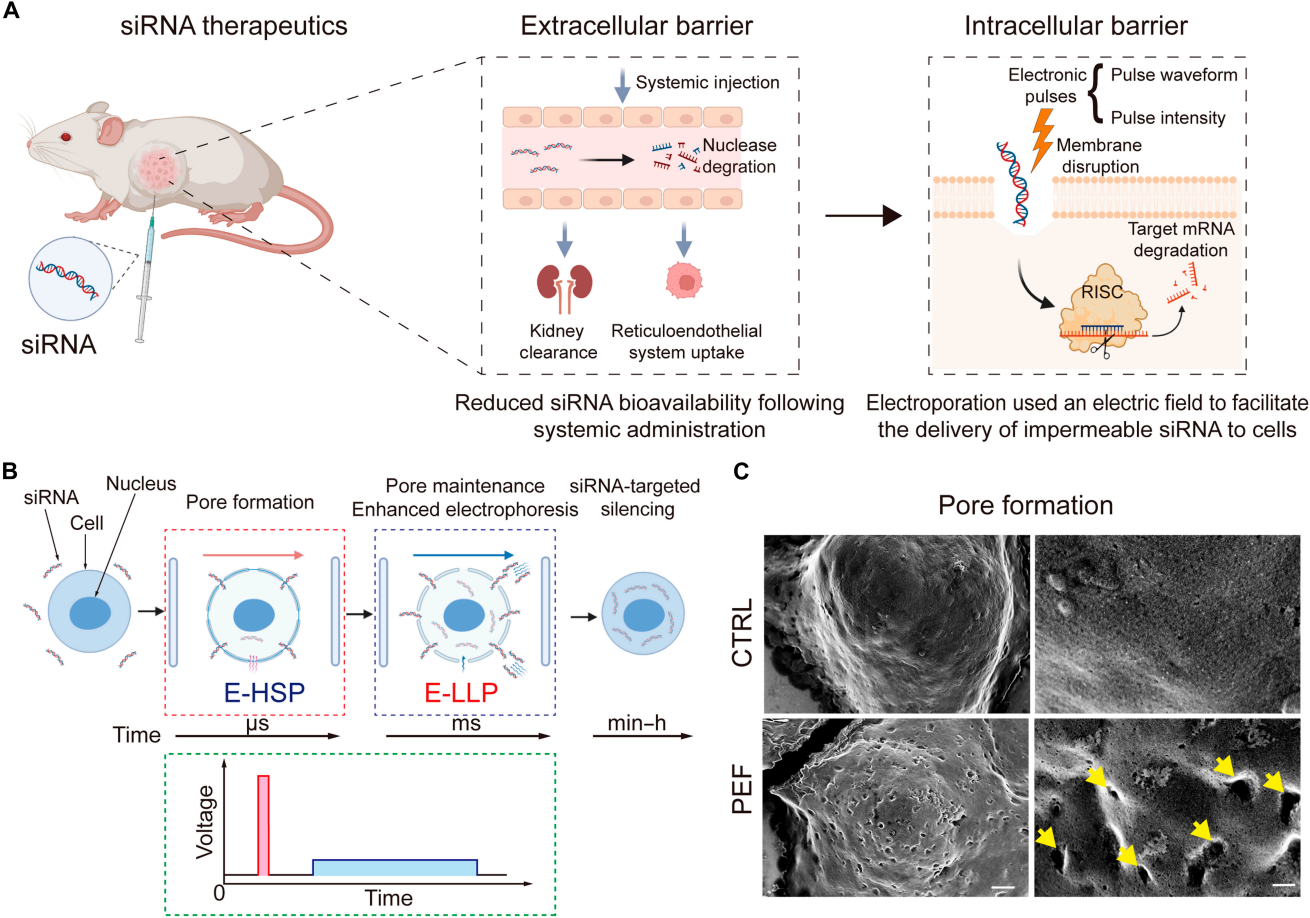
Overview of electroporation-mediated small interfering RNA (siRNA) delivery enhanced by pulse modulation. (A) Schematic illustration of siRNA delivery barriers, both extracellular and intracellular. (B) Steps in the transport of siRNA mediated by multiple-pulse modulation, combining effective electroporation and controllable pore dynamics. (C) Scanning electron microscopy of an MDA-MB-231 cell before (top) and after (bottom) exposure to an electric field (*E* = 750 V/cm, duration = 20 μs, frequency = 10 Hz, 50 pulses). Nano-sized pores (yellow arrows) caused by electroporation are visible on the cell membrane. Left scale bar, 1 μm. Right zoomed scale bar, 200 nm. mRNA, messenger RNA; RISC, RNA-induced silencing complex; HSP, high-voltage short-duration pulse; LLP, low-voltage long-duration pulse; PEF, pulsed electric field.

Challenges from the physical and biochemical environment hinder the delivery of siRNA into cells, while electroporation is one of the feasible approaches for improving its intracellular delivery (Fig. 1A). However, the demand for simple and robust electroporation-based platforms persists for lack of comprehensive insight into the role of pulse parameters in governing pore evolution. We propose PEP with multiple pulses, which decomposes the perforation process and delivery process (Fig. 1B). First, we verified the occurrence of membrane perforation with scanning electron microscopy (Fig. 1C). After exposure to fifty 750 V/cm × 20 μs × 10 Hz pulses, there were round hole-like shapes appearing on the membrane, exhibiting rough morphology compared to untreated cells. Then, we evaluated the effectiveness of different pulse modulation modes in facilitating intracellular delivery using a homemade pulse generator and elucidated the potential mechanisms from the perspectives of delivery dynamics, delivery efficiency, and pore evolution (Fig. S1).

### PEP enhanced intracellular delivery by regulating pore evolution dynamics

**Fig. 2.**
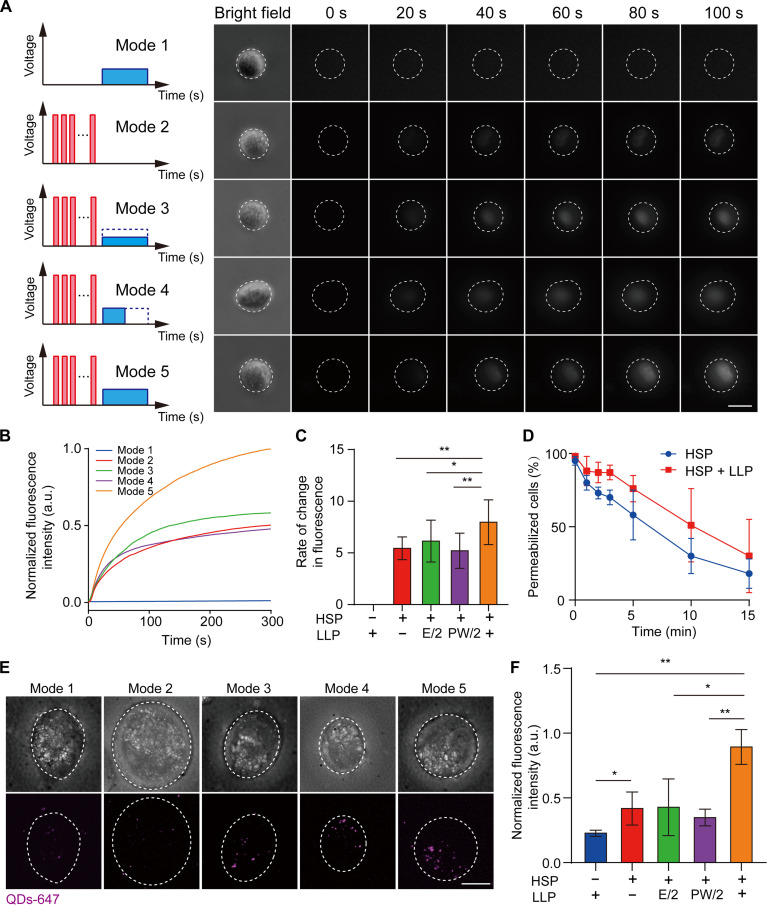
Effects of the pulse composition on delivery efficiency. (A) Time-lapse fluorescent imaging of propidium iodide (PI) after electroporation with 5 types of pulse waveforms. The first column shows the MDA-MB-231 cells under a bright field before electroporation. Mode 1, LLP (*E* = 75 V/cm, pulse width [PW] = 100 ms) alone. Mode 2, HSP (*E* = 750 V/cm, PW = 20 μs, frequency = 10 Hz, duration = 5 s). Mode 3, HSP + LLP_1_ (*E* = 37.5 V/cm, PW = 100 ms). Mode 4, HSP + LLP_2_ (*E* = 75 V/cm, PW = 50 ms). Mode 5, HSP + LLP_3_ (*E* = 75 V/cm, PW = 100 ms). Pulses were implemented at *t* = 0 s. Scale bar, 20 μm. (B) Quantification of intracellular fluorescence intensity pulsed with different pulse modulation modes as previously described. (C) Quantification of the fluorescence intensity change rate inside cells after exposure to different modulation modes (*n* = 50). (D) Dynamics of the cell membrane resealing process electroporated by HSP and HSP + LLP with regard to PI permeabilization (*n* = 3). (E) Electroporation-based intracellular delivery of quantum dots (QDs). Top, cell morphology in bright field. Bottom, representative fluorescence images of QDs-647 inside cells pulsed with different electric waveforms as previously described. Scale bar, 10 μm. (F) Normalized fluorescence intensity in cells after QD delivery using different pulse modulation modes (*n* = 50). Results are expressed as mean ± standard error of the mean (SEM) with 95% confidence interval (CI). In (C) and (F), significance was evaluated by one-way analysis of variance (ANOVA) for multiple comparisons; *P < 0.05 and **P < 0.01.

To elucidate the efficacy of multiple-pulse-facilitated delivery, we systematically altered the pulse compositions of multiple pulses to induce electroporation. The electric pulses could simply trigger cell permeabilization, which was necessary to gene transfer or molecule loading into cells [34]. We monitored the delivery efficiency of propidium iodide (PI) in MDA-MB-231 cells by measuring the mean fluorescence intensities (MFIs) in the cytoplasm. The pulse modulation modes included HSP, low-voltage long-duration pulse (LLP), HSP + LLP_1_, HSP + LLP_2_, and HSP + LLP_3_, which were designated in order as modes 1 to 5 (detailed parameters can be found in Table S1). The electric parameters were within the tolerable range of the cell, and the transmembrane potential was sufficient for electroporation to occur. The interval time between HSP and LLP was fixed at 0.1 s within the membrane relaxation time after electroporation. From the results of the MFI changes, we observed the delivery efficiency to be the highest for the HSP + LLP_3_ mode. The delivery efficiency using HSP + LLP_3_ was found to be 45% higher than that using a single HSP (Fig. 2A and B). By using LLPs, the amount of PI in the cytoplasm increased with pulse width and voltage amplitude, indicating that relatively long pulse widths and high voltage amplitudes were helpful for delivery. To empirically ascertain the incidence of delayed pore closure during electrotransfer, we examined the relationship between the uptake rate of exogenous molecules and the pulse mode employed from the change in fluorescence (Fig. 2C). Notably, HSP + LLP_3_ (mode 5) demonstrated superior efficacy in facilitating the entry of PI into the cells when compared to all other modulation modes. Further, we evaluated the membrane resealing dynamics between the groups treated with HSP + LLP_3_ (mode 5) and HSP (mode 2) by evaluating the PI permeabilization at different time points post-electric exposure. With the contraction of electropores on the membrane at various recovery times after electroporation, PI exhibited varying degrees of permeabilization. The permeabilization reached a plateau in both modes, and the half-life of permeabilization in HSP + LLP_3_ was extended for about 4 min compared to that in HSP (Fig. 2D). Rapid contraction of the electropores occurred within 1 min post-electroporation, and the recovery time spanned minutes [35]. Moreover, enhancement of intracellular delivery mediated by HSP + LLP_3_ was observed in the case of quantum dots (QDs). Experimental demonstrations using the multiple-pulse mode showed that HSP + LLP modulation in mode 5 was conducive to molecule intracellular delivery (Fig. 2E and F). Moreover, the total pulse duration also influenced electropermeabilization efficacy and cell viability, with permeabilization reaching a plateau and cell viability markedly deteriorating when the pulse number exceeded 50 at selected parameters (Fig. S2A). In the following sections, HSP + LLP_3_ was chosen as the optimal combination for PEP in the comparative experiments and bulk electroporation with a single pulse component (BEP) represented pulses composed of a single HV component.

### Elucidation of the regulatory effects of pore evolution by multiphysics field-coupled simulation

To understand the pore evolution and molecule intracellular delivery process into cells during electroporation, we used finite element simulation based on multiphysics field-coupled model (Fig. S1A). Müller et al. [36] reported that the time to reseal after pore formation increased with pressure and decreased with temperature by molecular dynamics simulations. It is worth noting that molecular dynamics simulations and their derivative approaches have provided valuable insights into pore state transitions [37], energy landscape evolution [38], prediction of electroporation sites [39], and conformational changes in membrane proteins [40] during electroporation. However, molecular dynamics simulations commonly employ idealized planar lipid bilayers and simplified pulse schemes, causing limited agreement with experimental observations [41]. Therefore, although prior studies have characterized molecular events within the lipid bilayer, the mechanisms by which pulse modulation strategies regulate pore formation and resealing remain to be further elucidated.

**Fig. 3.**
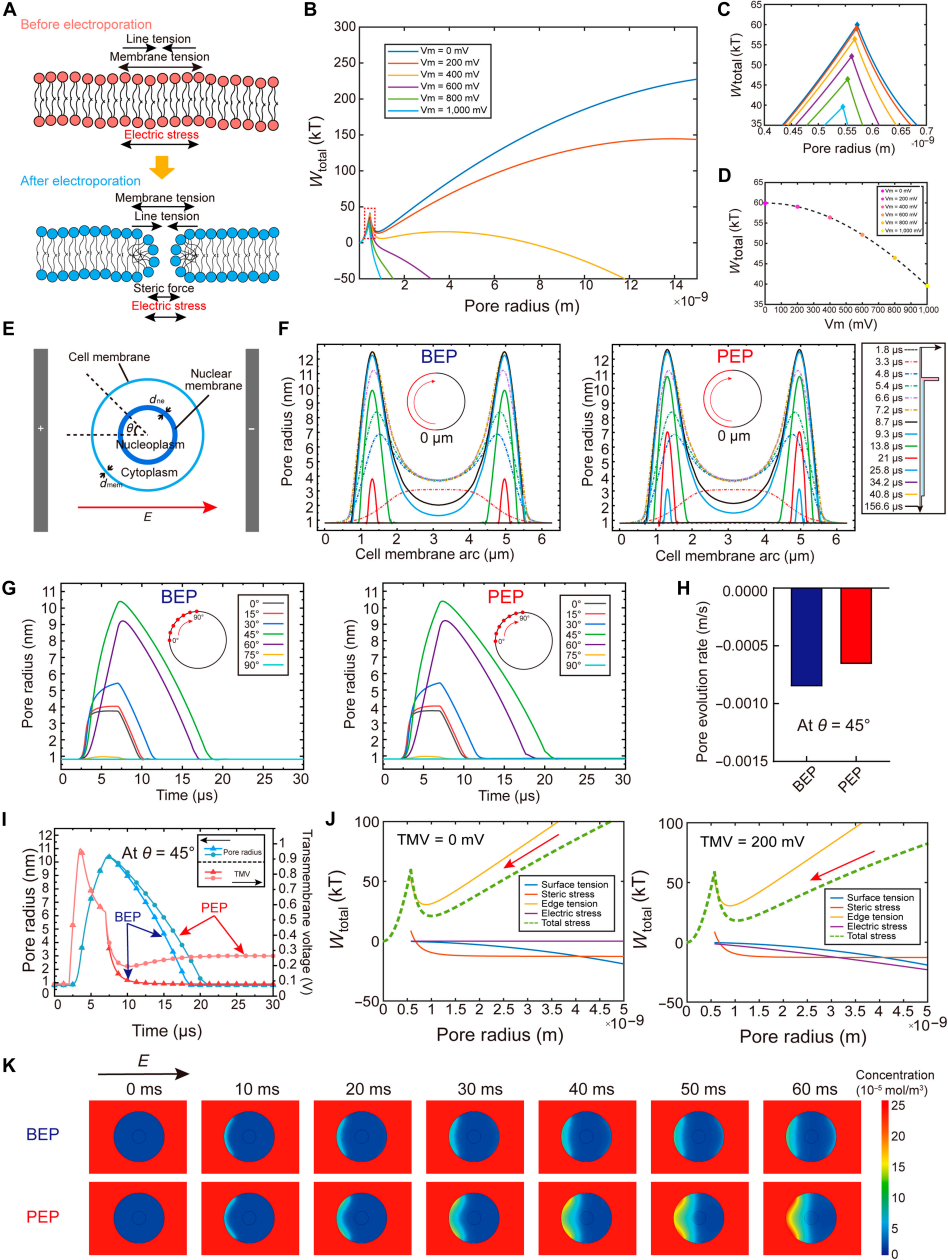
Numerical elucidation of pore evolution and the intracellular delivery enhancement mechanism by progressive electroporation. (A) Illustration of membrane conformation before and after electroporation. (B) Pore energy landscape with different transmembrane voltages. (C) Established transmembrane voltage decreases the energy barrier of pore electroporation. (D) Transitional pore energy from hydrophobic pores to hydrophilic pores decreases with the increase in transmembrane voltage. (E) Diagram of the double-layer dielectric cell model in the middle of 2 electrodes. (F) Evolution of the pore radius along the cell membrane with bulk electroporation with a single pulse component (BEP) and progressive electroporation (PEP). (G) Evolution of the pore radius at points along the cell membrane with polarization angles *θ* of 0°, 15°, 30°, 45°, 60°, 75°, and 90°. (H) Comparison of pore evolution rate with BEP and PEP at a polarization angle *θ* of 45°. (I) PEP retains the decrease in transmembrane voltage and delays pore resealing in comparison to BEP. (J) The transmembrane voltage influences energy variation during the resealing process of pores. The red arrow indicates the pore energy evolutional gradient with pore contraction. (K) Enhanced molecular transport of PI into electroporated cells with BEP and PEP at specific time points. TMV, transmembrane voltage.

Electric stimuli with 2 different pulse waveforms were applied to the classic double-shelled cell model in simulation as the representative of the suspended cell used in the experiments. The formation, expansion, and destruction of the “electropores” is governed by the Smoluchowski advection diffusion equation, which is derived upon a statistical mechanics framework [42]. Before electroporation, membrane energy fluctuation is balanced between membrane tension and line tension to maintain homeostasis [43]. With the implementation of an external electric field, the membrane energy landscape gets disturbed [44]. As the TMV increases, hydrophilic pores tend to appear, with contributions from both electric stress and the steric repulsion of lipid head groups (Fig. 3A). The critical TMV typically falls within the range of 0.2 to 1 V [16]. The electric field induces an increase in TMV, which causes a decrease in the transitional energy barrier of electroporation (Fig. 3B to D). Leveraging BEP and PEP exhibited different distributions of TMV along the cell membrane (Fig. S3A).

The simulation leveraged typical single- and multiple-pulse waveforms to theoretically reveal the effects of different pulse components on membrane electroporation. The cell model was assumed to be isotropic, and the electric field was oriented from left to right (Fig. 3E). The cell membrane underwent reversible electroporation featuring a pore distribution with a peak radius on both sides of the cell membrane with a polarization angle θ between 45° and 60° and a trough near the region facing the electric field (Fig. 3F). Both BEP and PEP produced similar pore radii in quantity and distribution. However, the trend in pore radius contraction differed between the 2 conditions. The modulated pulses retarded the disappearance of the pores, extending the closure time of individual pores at various angles along the cell membrane (Fig. 3G) and slowing the resealing rate of electropores (Fig. S3B). Furthermore, at *θ* = 45°, the slope of the pore resealing curve was calculated to reveal the delay of pore closure (Fig. 3H). With regard to the relationship between pore evolution and the establishment of TMV, the pore contraction happened almost simultaneously with the decay of TMV. At *θ* = 45°, PEP was found to hinder the decay of TMV at 200 mV in the simulation with prolonged pore resealing time (Fig. 3I). The existence of the remaining TMV might change the energy variation with a decreased gradient during pore resealing, as indicated with red arrows (Fig. 3J). The disturbance induced by LLP on pore energy is more directly manifested as a delayed relaxation of the pore free energy (Fig. S4). Furthermore, at elevated TMVs, the pore energy landscape no longer exhibits a metastable minimum in the hydrophilic regime (Fig. S3C), leading to uncontrolled pore expansion, inhibited spontaneous resealing, and ultimately irreversible cell death. It was suggested that LLP might provide electric stress and obstruct pore closure from the perspective of the force exerted on the pore [45]. Therefore, the simulation indicated that the multiple pulses did not affect the pore formation compared with a single type of pulse but rendered the pores open over an extended duration.

As further validation, we investigated the potential of modulated pulses on intracellular molecule transport. The creation of transient electropores has been widely acknowledged as the mechanism behind PEF-induced membrane permeabilization [46]. Here, we extended this model by integrating molecule transport governed by the Nernst–Planck equation into the traditional electroporation theoretical framework and investigated the differences in the delivery of small molecules such as PI), depending on the utilization of either BEP or PEP. For small molecules, diffusion across the concentration gradient prevails as the primary mechanism for cellular internalization throughout the existence of electropores [47]. In the simulations, leveraging multiple pulses, there were more small molecules delivered into the cell in terms of the intracellular PI concentration, with expanded distribution (Fig. 3K). If the molecules were charged, such as PI, which carries 2 positive charges, there would be an additional electrophoretic component that augmented intracellular distribution during the pulse [48]. Hence, it was suggested that the synergistic effect of regulating pore dynamics and electrophoresis enhanced the intracellular delivery of molecules.

### Investigation of pore resealing dynamics regulated by PEP and effects on cell viability

**Fig. 4.**
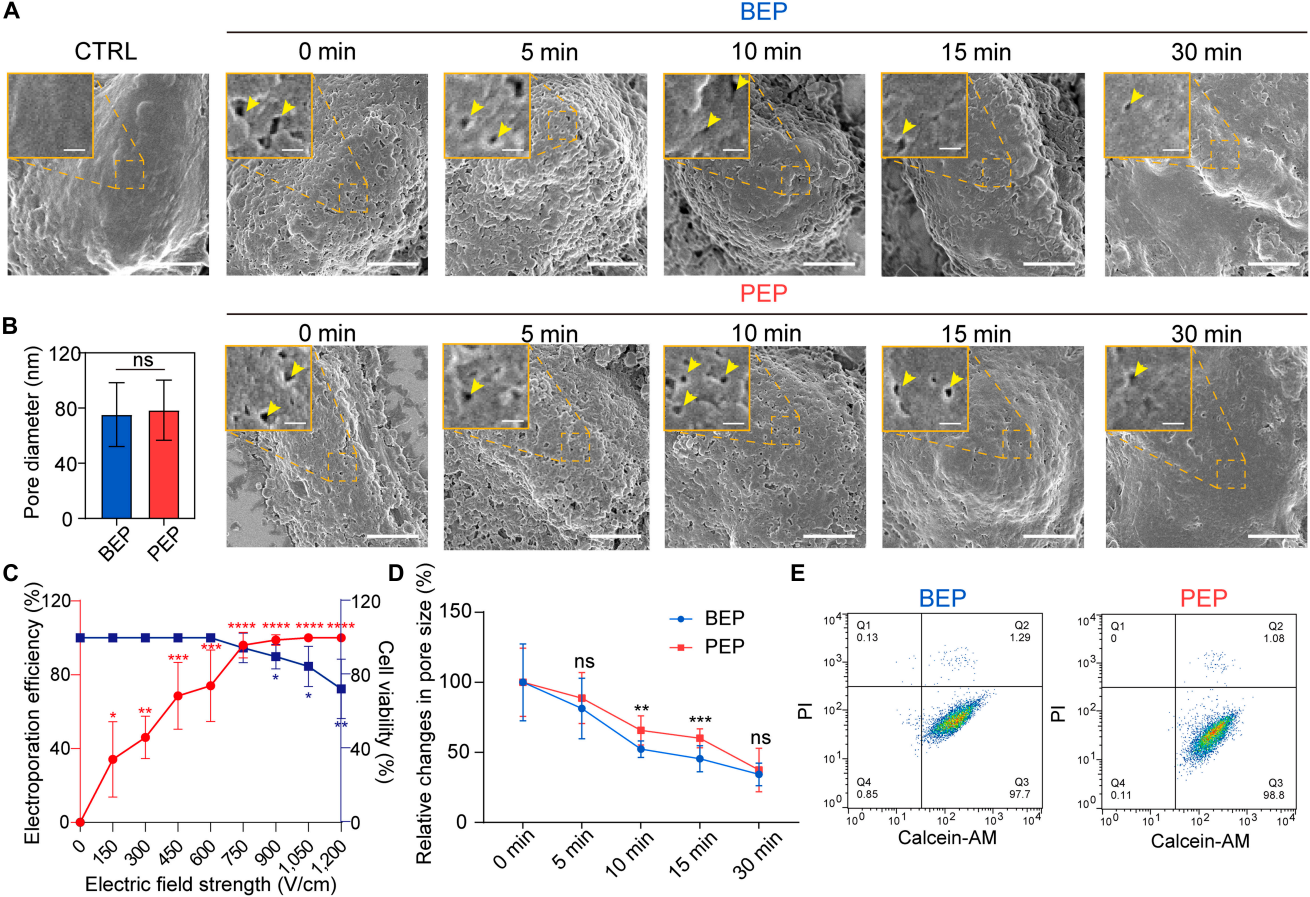
Pore evolution post-electroporation and cell viability analysis after BEP and PEP treatment. (A) Scanning electron microscopy images of cell membrane morphology at different recovery time points after exposure to BEP or PEP. Scale bar, 1 μm. Zoomed scale bar, 100 nm. (B) The immediate pore sizes created by BEP (*n* = 35) or PEP (*n* = 37). (C) The electroporation efficiency and 24-h cell viability after exposure to BEP with different electric field strengths (*n* = 3 for every trial). (D) Kinetics of pore resealing for BEP and PEP. (E) Analysis of cell viability by flow cytometry. Left, cells treated with BEP. Right, cells treated with PEP. The panels show staining with PI (dead cells are permeable to the dye) and calcein-AM (live cells hydrolyze the fluorogenic substrate). Results are expressed as mean ± SEM with a 95% CI. In (B), (C), and (D), significance was evaluated by an unpaired *t* test for individual BEP and PEP groups; *P < 0.05, **P < 0.01, ***P < 0.001, and ****P < 0.0001. ns, not significant.

To investigate the time-dependent variation of membrane after electroporation, scanning electron microscopy images of cells fixed after different recovery times post-electric stimulus were analyzed to reveal the morphological changes (Fig. 4A). Compared to untreated cells, electric stimulus roughened the membrane of cells, which showed a reversible trend in a time-dependent manner. Furthermore, there were no significant differences in pore formation after treatment with BEP or PEP with no differences in pore diameters (Fig. 4B).

We also investigated cell viability and electropermeabilization rate post-electric stimulation. Cell viability decreased as the electric field strength of the pulses increased for BEP. In contrast, when the electric field strength reached 750 V/cm, permeabilization was effective, with almost all cells permeable to PI (Fig. 4C). Consequently, treatment with BEP at 750 V/cm induced high perforation efficiency and maintained high cell viability. Moreover, the pulse width and electric field intensity of LLP did not affect the activity of cells (Fig. S5A and B).

Treating with BEP rendered a faster recovery time than treating with PEP (Fig. 4D), which indicated regulation of pore resealing dynamics by multiple pulses. Exposure to electric fields can induce membrane lipid oxidation, which has been proposed as a potential mechanism for sustained permeability [49,50]. However, after assaying the lipid oxidation after treatment with BEP, LLP, and PEP, no significant increase in lipid oxidation was observed (Fig. S6), indicating that lipid oxidation might not be the dominant contributing factor. Moreover, although cell swelling is a phenomenon that commonly accompanies membrane electroporation [51] and may involve the dynamic regulation of pore expansion and resealing [52], evaluation of the pulse modulation parameters used in this study revealed that in isotonic phosphate-buffered saline (PBS) buffer, the PEP strategy did not induce additional cell swelling over an observation period (10 min) far exceeding the pulse application time (Fig. S7). These results therefore suggest that cell swelling is not a primary factor during pore resealing regulated by PEP as well. Further, in the presence or absence of LLP, electric pulses with a field strength of 750 V/cm did not have a significant effect on cell viability (Fig. 4E). Taken together, these results showed that multiple pulses were effective in prolonging the pore closure process to an appropriate degree without affecting cell viability, which also experimentally validated the previous simulation results and explained how multiple pulses improved delivery efficiency.

### Efficacy of PEP in serving siRNA electrotransfer in vitro

To gain a deeper insight into the mechanism of multiple-pulse electrotransfer, we carried out a comparative study among 2 widely used siRNA transfection technologies: electrotransfection and lipofection (using RNAiMAX from Thermo Fisher). In order to determine the efficiency of siRNA transfection, we used green fluorescent protein (GFP) siRNA targeting the messenger RNA of GFP constitutively expressed in MDA-MB-231-GFP cells. The optimal electric parameters for electroporation and the associated electrotransfer were determined by monitoring both the penetration of PI into cells and cell viability.

**Fig. 5.**
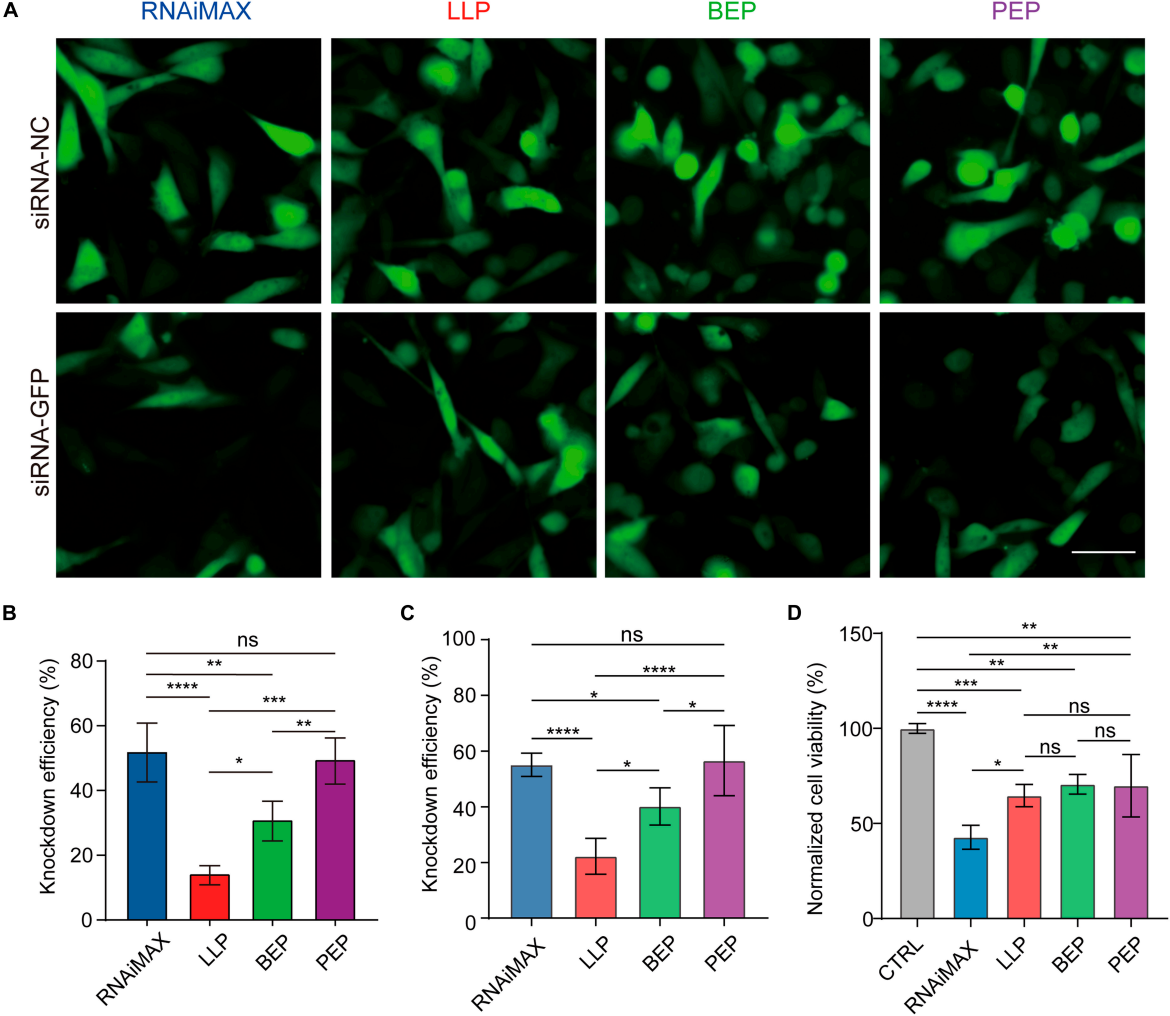
Comparison of the knockdown efficiency and cellular activity after targeted siRNA electrotransfection and lipofection. (A and B) siRNA knockdown experiment with MDA-MB-231-GFP cells. (A) Representative fluorescence images of green fluorescent protein (GFP) expression inside MDA-MB-231-GFP cells after nontargeted or targeted siRNA delivery with the transfection reagent RNAiMAX or electroporation with different waveforms (LLP, BEP, and PEP). Top: noncoding siRNA (siRNA-NC), which does not silence GFP expression. Bottom: siRNA-GFP, which specifically targets GFP expression. Scale bar, 50 μm. (B) Quantification of GFP expression and transfection efficiency after siRNA delivery (*n* = 4). (C and D) siRNA knockdown experiment with MDA-MB-231-Luciferase cells. (C) Quantification of luciferase expression and transfection efficiency after siRNA delivery by the transfection reagent RNAiMAX or electroporation with different pulse waveforms (LLP, BEP, and PEP) (*n* = 5). (D) Effects on cell viability at 48 h after siRNA transfection by the transfection reagent RNAiMAX or electroporation with different waveform modulations, normalized to the control group incubated with siRNA alone (*n* = 4). Results are expressed as mean ± SEM with 95% CI. In (B), (C), and (D), significance was evaluated by one-way ANOVA for multiple comparisons; *P < 0.05, **P < 0.01, ***P < 0.001, and ****P < 0.0001.

We quantified the delivery efficiency of siRNA in the MDA-MB-231-GFP cells by comparing the residual GFP expression in the cytoplasm after knockdown by siRNA targeting GFP (Fig. 5A). The knockdown efficiency under various electric fields was demonstrated with a relative value proportional to the treatment of negative control siRNA delivery (Fig. 5B). The expression of GFP was hardly affected in all controls. The MFI of the GFP-positive cells would not be modified despite the successful electrotransfer of an unrelated siRNA. Meanwhile, with the siRNA targeting GFP transfected into cells by lipofection, a significant decrease in GFP-positive cells occurred within 48 h after treatment. Surprisingly, a reduction in GFP expression was observed with PEP-mediated siRNA electrotransfer, which was equivalent to the effect of lipofection (47%). In contrast, the siRNA electrotransfer induced by LLP or BEP did not show a significant decrease in GFP expression. To further compare the effects of these 2 protocols on cell viability, we used PEP with multiple electric field strengths (600, 750, and 900 V/cm) for comparisons with RNAiMAX (Fig. S5C). The trend showed that as the voltage increased, the efficiency of siRNA electrotransfer increased, but the cell viability also decreased, and remarkably, at 600 and 750 V, only about 50% of cells died during transfection. However, RNAiMAX used at the dose recommended by the manufacturer’s protocol caused a significant decrease in cell viability. Taken together, multiple pulses with high voltage amplitudes within a certain range maintained cell viability and also had an excellent electrotransfer efficiency.

The same procedure was conducted for siRNA transfection in MDA-MB-231-Luciferase cells (stably expressing firefly luciferase) to verify the knockdown efficiency of electroporation-mediated siRNA transfection as well (Fig. 5C). Multiple pulses realized comparable knockdown efficiency (57%) versus RNAiMAX transfection (55%). BEP had a higher knockdown efficiency than LLP (40% vs. 22%) but lower than that of PEP. Cell viability at 48 h after siRNA transfection using LLP, BEP, PEP, or RNAiMAX was also compared by 3-(4,5-dimethylthiazol-2-yl)-2,5-diphenyltetrazolium bromide (MTT) assay (Fig. 5D). siRNA transfection by LLP, BEP, and PEP showed significantly higher viability (65%, 71%, and 70%, respectively) compared to the chemical RNAiMAX method (43%).

### Improvement of siRNA knockdown efficiency in vivo by PEP application

In vivo electrotransfection has been recognized as an effective technique for gene editing within tissues [16,53]. Although it has been shown that tissue such as retinal cells can be readily introduced into neonatal mice by electroporation, the delivery efficiency using traditional single pulses was low, and further improvement of transfection conditions was needed [54]. Prior comparative studies have demonstrated the advantages of combining HV and LV pulses for in vivo nucleic acid delivery, including DNA, although systematic evaluations of tissue damage and biosafety remain limited. André et al. [25] reported the superiority of HV and LV pulse combinations for DNA electrotransfer in muscle, liver, tumor, and skin. Bureau et al. [55] systematically evaluated the delivery efficacy of HV and LV pulses at varying field strengths, as well as their combinations, using series of identical square-wave pulses, and demonstrated that combined HV + LV pulses outperform either HV or LV alone for in vivo transfection of skeletal muscle. Pavšelj and Préat [56] confirmed that the combination of one HV and one LV pulse was more efficient than HV or LV pulses alone in delivering plasmid DNA into the skin with tolerable adverse effects. Since we conducted comprehensive comparisons of different pulse combinations across various molecular cargos based on quantitative fluorescence analyses in in vitro experiments, establishing a foundation for enhanced intracellular delivery efficacy by PEP, the present study placed particular emphasis on the in vivo applicability and safety of the PEP strategy. A subcutaneous MDA-MB-231-Luciferase xenografted murine tumor model was established to validate whether in vivo electroporation by multiple pulses efficiently mediated siRNA delivery into tumor tissue and suppressed the expression of targeted genes (Fig. S8), with the device setup demonstrated in Fig. S9.

**Fig. 6.**
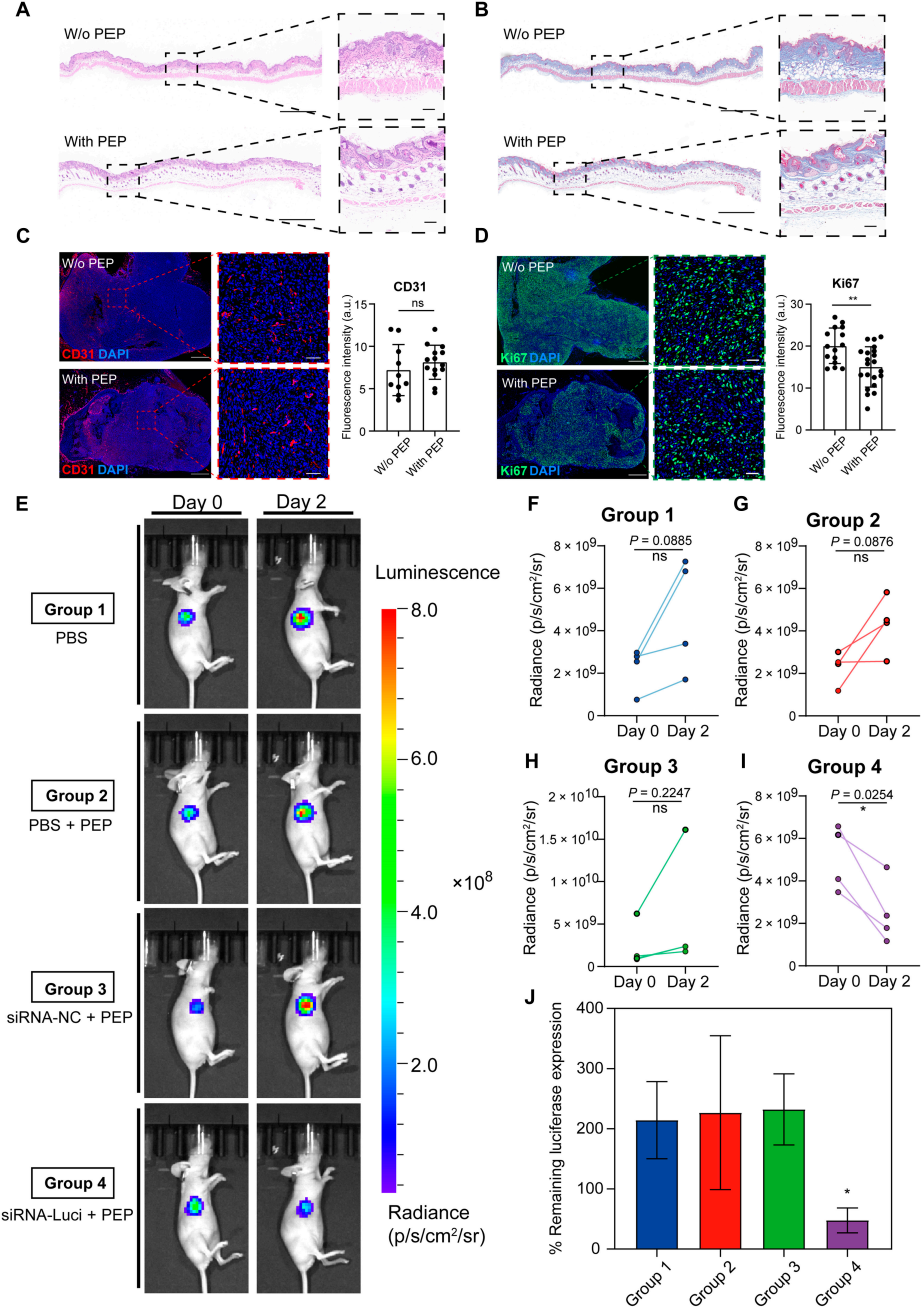
siRNA knockdown efficiency in vivo by PEP implementation. (A) Hematoxylin and eosin (H&E) staining analysis of skin with or without PEP treatment. Scale bar in global area, 1 mm. Scale bar in zoomed area, 100 μm. (B) Masson staining analysis of skin with or without PEP treatment. Scale bar in global area, 1 mm. Scale bar in zoomed area, 100 μm. (C and D) Representative in situ immunofluorescence images and quantification analysis of tumors with or without PEP treatment. CD31 staining (C); Ki67 staining (D). Scale bar in global area, 800 μm. Scale bar in zoomed area, 50 μm. (E) Luciferase expression before (day 0) and after (day 2) targeted siRNA electrotransfection in vivo in subcutaneous tumors. (F to I) Quantification of the remaining luciferase expression in vivo in tumors at day 2 compared to that at day 0 for groups treated with PBS injection (F), phosphate-buffered saline (PBS) injection with PEP (G), siRNA-NC (not targeting any gene) injection with PEP, (H) and siRNA-Luci (targeting firefly luciferase) injection with PEP (I). (J) Quantification of normalized remaining luciferase expression in vivo in tumors at day 2 compared to that day 0. Results are expressed as mean ± SEM with 95% CI. In (C) and (D), significance was evaluated by 2 unpaired *t* tests; *P < 0.05 and **P < 0.01. In (F) to (I), significance was evaluated by 2 paired *t* tests; *P < 0.05. In (J), significance was evaluated by one-way ANOVA for multiple comparisons; *P < 0.05. DAPI, 4′,6-diamidino-2-phenylindole.

To assess the feasibility of PEP in in vivo siRNA transfection, we firstly evaluated the influence of PEF exposure at selected parameters on normal skin and tumors. The hematoxylin and eosin and Masson staining results demonstrated that there were no significant effects on the histological structure of normal skin (Fig. 6A) and the distribution of collagen fibers (Fig. 6B). Furthermore, PEP exerted minimal influence on angiogenesis (Fig. 6C), while it significantly suppressed cellular proliferation (Fig. 6D), which likely underlies the localized reduction in cell density observed in tissue sections following PEP treatment.

In consideration of the knockdown efficiency, the luciferase activities posttreatment exhibited a significant increase for groups 1, 2, and 3 while luciferase radiance consistently decreased with targeted siRNA delivery for group 4 (Fig. 6E to I). Quantification of normalized remaining luciferase expression revealed that the intensity increased by 114.3%, 126.7%, and 132.3% for groups 1, 2, and 3, respectively, compared with those before treatment. Since the luciferase gene was stably integrated into the genome of MDA-MB-231 cells, its expression in every single cell should remain at a relatively stable level if the tumor cells were not stressed and were unaffected by electroporation. This suggested that the proliferation of tumor cells should have resulted in an increase in luciferase expression in this time frame. In contrast, group 4 luciferase expression post-electroporation was down-regulated with a 52.3% inhibition efficiency compared with the luciferase levels before electroporation treatment (Fig. 6J). This result demonstrated that the anti-luciferase siRNAs completely erased any increase in luciferase expression due to the proliferation of tumor cells and further lowered the existing luciferase levels.

**Fig. 7.**
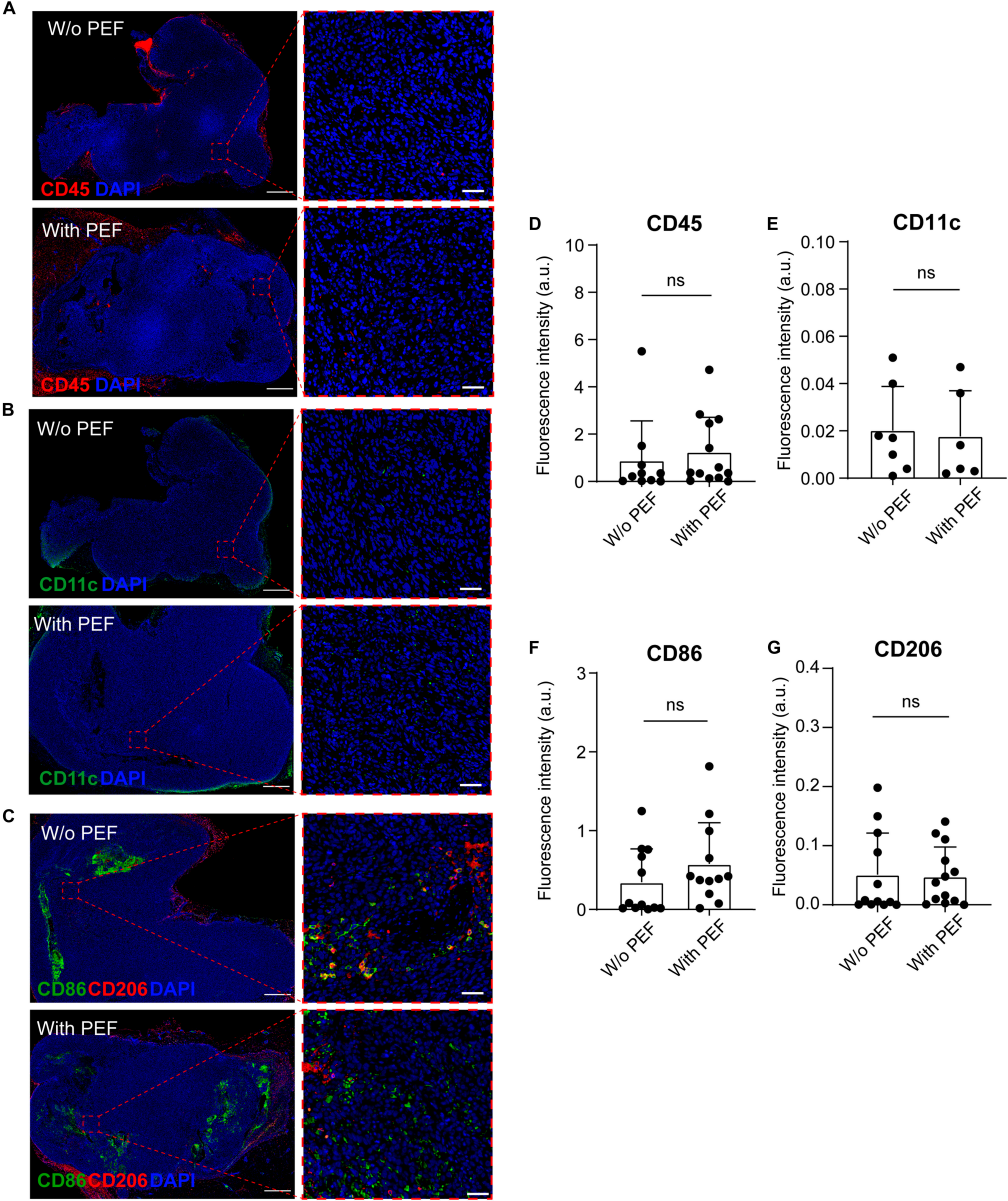
Immunofluorescence analysis of anti-tumor immunomodulation with or without exposure to PEP. (A to D) Representative in situ immunofluorescence images of tumors with or without PEP treatment. CD45 staining (A), CD11c staining (B), and CD86/CD206 staining (C). Scale bar in global area, 800 μm. Scale bar in zoomed area, 50 μm. (D to G) Quantification of the immunofluorescence intensity of tumors with or without PEP treatment. Results are expressed as mean ± SEM with 95% CI. In (D) to (G), significance was evaluated by 2 unpaired *t* tests.

Further, it has been reported that electric stimulus can induce anti-tumor immunomodulation [53,57,58]. We investigated the common immune cells clustered or immersed in the tumors by immunofluorescence analysis. Although a slight increase was found in the markers of leukocytes (CD45) and dendritic cells (CD11c) by PEP treatment, there was no significance between the groups with or without PEP treatment (Fig. 7A, B, D, and E). Similar trends were found in the aspect of the polarization of macrophages (M1 and M2) (Fig. 7C, F, and G). Although electric stimulus might involve the inflammatory response and render the local apoptosis of tumor cells, the immune modulation can be activated to different degrees depending on the electric parameters [57]. Taken together, we validated the feasibility of this combined pulse strategy for siRNA delivery both in vitro and in vivo, which may contribute to siRNA therapeutic applications in the future.

## Discussion

A PEF can profoundly trigger the conformation of the membrane in a transient manner, which renders intracellular delivery. Compared with other delivery approaches, electroporation-based strategies offer notable advantages, including broad applicability, relatively high efficiency, and the preservation of cellular structures and functions [8,12]. The integration with microfluidic chips can, under certain conditions, mitigate cell mortality and enable visualization at the single-cell level. However, this benefit is offset by the increased complexity of device fabrication and operation [59]. Similarly, micro/nanostructure-based techniques, such as microneedles, allow for precise dosage control with minimal cellular damage, but their invasive nature, high cost, dependence on biocompatible materials, and intricate operational requirements pose critical challenges to their broader application [12,60]. To enhance the efficacy of electroporation for intracellular delivery, pulse modulation can play a pivotal role without imposing additional demands on device development.

By modulating the pulse composition, electroporation-mediated intracellular delivery can be improved to adapt to the demands of different applications. The pore morphology after electroporation was firstly resolved by fast-freezing scanning electron microscopy in 1990 [61]. Shallow evaginations with no discontinuity and flat pores with no sharp curvature were observed on the membrane after electroporation. Although optical methods by virtue of fluorescent probes can provide resolved and indirect information of the electropores, such a method is limited to revealing the phenomena at the bottom of the cells, while the adhesion of the membrane to the substrate may interfere with the formation of pores [62]. Moreover, an optical method cannot image the structure of pores directly through resolving the locations of individual pores and is inevitably constrained by the diffraction limit [63]. Since the morphology of electropores for various types of cells have been reported via advancements in electron microscopy technology [64,65], scanning electron microscopy may help reveal the existence and evolutionary trends of electroporated pores at the cellular level.

Our results indicated that cell electroporation induces structural alterations in the membrane, giving rise to morphological characteristics distinct from those observed in untreated cells (Fig. 4A) [18]. Previous theoretical modeling reported a toroidal shape in pore construction as well [66]. Furthermore, we provided an insight into the theoretical mechanisms behind the pore resealing dynamics regulated by electric pulse modulation combining HSP and LLP, which could also be supported by the morphological characterization of the membrane. It has to be mentioned that combined pulses were commonly considered to improve the electrotransfer of charged large molecules into cells with electrophoresis [29]. Since conservative millisecond pulse parameters were employed to minimize adverse effects like electrolysis and heating for preserving cell viability (Fig. S10) [67], the electrophoretic effect might be unable to exert a lasting influence on molecular transport in that it persisted only during the period of electric field exposure. However, apart from the regulation of the motion of molecules, a modulated electric field may have an effect on pore evolution as well since the fate of pores is strongly dependent on the selection of electric parameters [21]. Here, we shed light on the prolonged resealing of electropores induced by the PEP strategy. With the decoupling of membrane electroporation and intracellular delivery, effective electroporation was realized with a shortened HSP duration for reduced cell damage and efficient delivery was realized with the combination of LLP immediately after HSP in a progressive manner.

The simulation demonstrated the delay of the pore resealing process in a polarization-angle-dependent manner when applying PEP (Fig. 3G) ranging within the typical evolution time for simulation [35]. In this study, treating with BEP or PEP created similar-sized pores on the membrane immediately after electric stimulation. However, multiple pulses profoundly interfered with the resealing process of electroporated pores in experimental conditions, with an approximately 5-min delay in pore recovery to initial state (Fig. 4D). Combined with the experimental results of PI permeation at different time points, PEP strategy rendered delayed pore closure for more molecules to enter the cell (Fig. 3B to D). Moreover, the universality of the PEP strategy in enhancing intracellular delivery and pore regulation was further confirmed on NIH 3T3 and HeLa cells (Fig. S11). Given that distinct membrane properties among different cell types influence their response to external electric fields, the application of the PEP strategy can be further optimized through careful parameter selection [68]. Nonetheless, modulated electric pulses facilitating the maintenance of the pore was verified by simulation and experimental results.

To better understand the mechanism, we investigated the changes in pore energy landscape, which assumed an important role in pore evolution. As membrane electroporation is a metastable process governed by the energetic balance among membrane tension, pore edge tension, electric stress, and the repulsive forces of lipid head groups, different components of modulated pulses can impose different effects on the pore energy landscape, thus influencing pore evolution [44]. Before electroporation, an external electric field charged the membrane and rearranged the ions around the membrane [69]. Once above the breakdown threshold (about 1 V), membrane electroporation occurred with a sudden decrease in TMV. The energy barrier for the formation of hydrophilic pores got decreased with the increase in TMV, which indicated the disturbed balance in the energy landscape with the external electric field (Fig. 3B and C). It has been widely reported that pore resealing is influenced by the edge line tension of the pores [70–72]. Although the establishment of TMV polarized the arrangement of the membrane lipid bilayer, there was no evidence supporting the regulation of TMV on pore edge tension [71]. Nonetheless, the existence of TMV indicated a state of electric charge arrangement around the membrane, which might facilitate the movement and transport of ions and molecules across the membrane in the form of flux. Meanwhile, after the withdrawal of the electric field, the TMV decays, accompanied by pore resealing. Recently, the membrane electroporation process was refined theoretically with transitional states in which water molecules infiltrated the interstitial spaces between lipid molecules in molecular simulation [38]. Hence, the retained TMV might be related to the unstable pore state and suggested the gradual withdrawal of fluid from the lipid bilayers, thus influencing pore evolution [45]. Although the current simulations were conducted using a simplified cell model with assumptions of cellular homogeneity, isotropic membrane properties, and idealized pore dynamics, as commonly employed in electroporation studies [73], they served as a complementary tool to experimental observations, supporting the conceptual framework of PEP and providing mechanistic insights into the regulation of pore expansion, resealing dynamics, and subsequent molecular transport by combined pulses [74–76]. To better simulate real-life cell conditions, more efforts should be directed toward incorporating factors such as irregular cell morphology [77], cellular deformation [78], and complex pore dynamics into a more refined model, grounded on accurately measured data, which constitutes an important outlook for theoretical studies of electroporation [79]. It is worth noting that the pores must reseal; otherwise, the cell might experience severe damage. The maintenance of the pores caused by PEP was moderate without undesired damage to cell viability.

Different kinds of molecules were used to confirm the universality of PEP to evaluate the enhanced efficacy of multiple pulses in practical intracellular delivery applications. Time-lapse imaging of the fluorescent dye PI showed a faster delivery rate using multiple pulses compared with other modulation modes (Fig. 2A and B). The delivery efficiency of QDs was also increased by multiple combined pulses (Fig. 2F). Further, multiple pulses showed an excellent delivery performance for siRNA with improved cell viability and considerable transfection efficiency compared to the Lipofectamine method (Fig. 5). The applicability and effectiveness of the proposed pulse modulation was verified in in vivo transfection of siRNA to knock down the targeted gene expression in subcutaneous tumors without side effects evaluated from a histological perspective after 2 d (Fig. 6). However, we recognize that this time frame is insufficient to evaluate the duration of gene silencing effects, potential delayed tissue responses, and adaptive immune responses that may develop over days to weeks. Although longitudinal studies extending to 2 to 4 weeks posttreatment, comprehensive histopathological and biochemical analysis of tissue response, and systematic evaluation of immune responses through cytokine profiling and lymphocyte analysis remain to be conducted for practical clinical implementation, our study has confirmed that PEP can act as an effective tool in improving the delivery efficiency of different molecules, which provides a foundation for these essential long-term safety and efficacy studies and holds potentials in cellular monitoring [65,80] and cell therapy [81,82]. Further, although LLP with a low electric field has been assumed to be not effective in permeabilization [25] and incubation with siRNA alone cannot bring about effective transfection as well, the implementation of LLP alone can achieve about 20% delivery efficiency for siRNA (Fig. 5A and B) and also caused a low uptake of QDs (Fig. 3F). This may be attributed to the activation of cellular active uptake processes, such as endocytosis, post-exposure to external electric stress [83]. In our study, we demonstrated the involvement of lysosomal escape employing carboxyfluorescein (FAM)-labeled siRNA after 2-h incubation with RNAiMAX reagent or post-PEP exposure (Fig. S12). The results indicated that although lysosomal pathways get involved in PEP-mediated siRNA delivery, PEP renders a significant decrease in the colocalization of lysosomes and FAM-siRNA, which may be due to direct cytosol delivery or enhanced lysosomal escape [33]. Therefore, the potential role of an applied electric stimulus in tuning cellular endocytosis should be further recognized in future work [84].

In addition, although pore dynamics had been investigated employing fluorescent probes [62] and other indirect electrophysiologic methods [80], instant and direct investigation of the electropores was not feasible in our study in that fixation with 2.5% glutaraldehyde needs a specific time for full fixation of cells, during which membrane resealing may happen. Therefore, this method hardly obtains precise pore sizes instantly at the occurrence of membrane electroporation. Considering that pore dynamics were controlled by comprehensive forces such as membrane tension and electric stress in theoretical modeling, we could not evaluate the real-time fluctuation by experimental methods. Moreover, in vivo observation of pore formation and the transferring process of molecules has also been limited due to the lack of advanced detecting techniques. In the future, by means of traction force microscopy and sequencing techniques, biomechanical and gene regulation induced by pulse modulation in cell electroporation may promote a better understanding of PEP-mediated intracellular delivery. Further, since electric field distribution decays with the increase in depth, the effective depth of an externally applied electric field ranges within micrometers to millimeters by transdermal electropermeabilization [85]. To further facilitate delivery into regions deeply embedded within tissue, invasive electrodes or microneedle-based devices can be integrated into the system to enhance delivery efficiency and expand the effective treatment depth [86].

## Conclusion

In this study, we presented PEP, which combines HV microsecond pulses (HSP) and LV millisecond pulses (LLP) to regulate pore closure dynamics for enhanced intracellular delivery. HSP with shortened duration and total treatment time effectively induced membrane electroporation while preserving cell viability, whereas LLP perturbed pore expansion and resealing by modulating TMV decay and pore energy fluctuations, thereby extending the pore lifetime. Simulations based on an established electroporation model of the pore energy landscape, together with experimental results, demonstrated that PEP delayed pore closure compared to BEP, promoting a prolonged delivery window for diverse molecules. PEP outperformed chemical lipofection in RNA interference efficiency (57% vs. 55%) while improving cell viability (70% vs. 43%) for siRNA delivery and reduced electrical energy consumption by 17%. These findings highlighted that regulating pore resealing dynamics by pulse modulation is a crucial mechanism for enhancing intracellular delivery efficiency while minimizing cytotoxicity. In summary, PEP provides a mechanistic and practical framework for improving transcellular transport via pulse modulation and offers potential for the development of next-generation, energy-efficient delivery systems.

## Materials and Methods

### Numerical simulation

Numerical simulation was conducted with the software COMSOL 5.4 Multiphysics (Stockholm, Sweden) based on the double-shelled cell model. The structural components in the model were assumed homogeneous, and parameters for individual structures were set according to respective dielectric properties. The cell model was 20 μm in diameter, similar to the size of human breast cancer cells (MDA-MB-231), and was placed in the center of 2 electrodes 1 mm apart. The multiphysics field model was built according to the computational framework [73] coupling electric field distribution, pore evolution, and molecule delivery process (Fig. S1A).

Electric field distribution can be calculated by solving the Laplace equation, which is in the form of Eq. (1):

$$\nabla \cdot \left[\left(\sigma +\varepsilon_{0}\varepsilon_{r}\frac{\partial }{\partial t}\right)\nabla \phi_{m}\right]=0,$$ (1)

The transmembrane potential $\phi_{m}$ can be obtained by the differentiation of the potentials inside and outside the cell membrane: $\phi_{m}=\phi_{i}-\phi_{o}$ [16].

Pore creation and destruction are relevant to the pore energy landscape and can be described by Eq. (2) [16,87]:

$$\begin{array}{c} \begin{array}{c} \frac{{\mathrm{dr}}_{p}}{\mathrm{dt}}=\left(D_{p}/\mathrm{kT}\right)\left(\frac{{\phi_{m}}^{2}F_{\max}}{1+r_{h}/\left(r_{p}+r_{t}\right)}+4\beta {\left(\frac{r^{*}}{r_{p}}\right)}^{4}\frac{1}{r_{p}}-2\pi \gamma +2\pi \delta_{\mathrm{eff}}r_{p}\right), \end{array} \end{array}$$ (2)

where $r_{p}$ is the pore radius and $D_{p}$ is the pore diffusion coefficient in the pore radius space. k is the Boltzmann constant, and T is the temperature. $F_{\max}$ is the maximum electric force, and $r_{t}$ and $r_{h}$ are constants for the advection velocity. β is the steric repulsion energy, $r^{*}$ is the minimum radius of the hydrophilic pores, and γ is the edge energy. $\delta_{\mathrm{eff}}$ is the effective tension coefficient of the cell membrane. The evolution of pores on the cell membrane during electroporation is thought to be related to the synergistic effect of the surface tension of the cell membrane, repulsive force of lipid heads, and edge line tension on the pore perimeter, composing the energy landscape W of the pore.

To couple the molecule transport into membrane electroporation, the membrane diffusion coefficient of particles was introduced as follows [76]:

$$D_{m}=0.01\times D_{c,\mathrm{dif}}\left(1-p\right)+D_{c,\mathrm{dif}}p,$$ (3)

where $p=\mathrm{N\pi}{r_{p}}^{2}$, indicating the permeability of the membrane. $D_{c,\mathrm{dif}}$ is the diffusion coefficient in the medium; $q_{e}$ and $z_{c}$ refer to the net charge and molecular charges, respectively; and $N_{A}$ is the Avogadro constant. Molecule transport can be described by the Nernst–Planck equation, which consists of the free diffusion component and the electrophoretic component as Eq. (4) [73]:

$$J_{c}=-D_{c,\mathrm{dif}}\nabla n_{c}-\frac{D_{c,\mathrm{dif}}q_{e}\left|z_{c}\right|}{\mathrm{kT}}n_{c}E,$$ (4)

where $J_{c}$ refers to the specific molecule flux and $n_{c}$ is the molecule concentration. To unveil the individual effects of 2 pulse components in PEP, a single HSP (750 V/cm, 5 μs) and a single LLP (75 V/cm, 100 ms) with no interval time were applied in the simulation. Detailed description of modeling methods and parameters can be found in the Supplementary Materials and Table S2.

### Device fabrication and experimental setup

A solid-state Marx generator (SSMG) based on capacitors charged in parallel and discharged in series was used to generate modulated rectangular pulse waveforms [88,89]. The waveform of the output pulses was adjusted by the changed number of series capacitors. A typical unipolar SSMG consisted of a positive multiple-pulse topology (Fig. S1C). In this part of the experiment, SSMGs with positive pulse topologies were stacked and a novel unipolar multiple-pulse topology was proposed (Fig. S1D). The setup of the PEF stimulation chamber consisted of Pt electrodes secured to the top lid of a cell culture chamber fitted on a standard 35-mm culture plate that facilitated handling and sterilization and reduced medium evaporation. A pair of electrodes with dimensions 20 mm × 20 mm × 1 mm, separated by a distance of 10 mm, was passed through the pre-drilled cover of the 35-mm culture plate and connected to a self-made pulse generator. The pulse generator was programmed to generate square monophasic waves consisting of HSP accompanied by LLP delivered to the entire setup. The waveforms were monitored by a P6015A probe (Tektronix, Ltd.) and an MS0-X3204 oscilloscope (bandwidth 200 MHz, Agilent, Ltd.) (Fig. S1B).

### Cell culture and molecules

The MDA-MB-231 cell line was provided by the Cell Bank/Stem Cell Bank, Chinese Academy of Sciences (Shanghai, China). Cells were routinely cultured in T25 flasks containing 5 ml of Dulbecco’s modified Eagle medium culture medium supplemented with 10% fetal bovine serum (Invitrogen) at 37 °C in a humidified atmosphere containing 5% CO_2_. MDA-MB-231 cells expressing green fluorescent protein (GFP) (MDA-MB-231-GFP) and luciferase (MDA-MB-231-Luci) were obtained after retroviral transduction and maintained in culture according to a previously described protocol [90]. PI was purchased from Sangon (Shanghai, China). QDs-647 were a gift from Prof. Pang’s research group (Wuhan, China), which were synthesized and characterized as reported by Zhang et al. [91]. GFP siRNA, with the target sequence 5′-GCA CCA UCU UCU UCA AGG A-3′, and luciferase siRNA, with the target sequence 5′-CUU ACG CUG AGU ACU UCG A-3′, were used in the experiments. These sequences were adopted from published studies [92]. FAM-labeled nontarget siRNAs were purchased from GenePharma (Shanghai, China). The LysoPrime Deep Red probe was purchased from Dojindo Laboratory (Beijing, China).

### In vitro electroporation

Electroporation was performed using a self-built device, which delivered arbitrary square-wave electric pulses. To avoid the electric drift of the cells during pulsation, adherent cells were grown on 6-well plates or 35-mm dishes for fluorescence microscopic observations. The electroporation chamber was designed using a pair of parallel electrodes, with a 1-cm interval. The electrodes were connected to the pulse voltage generator, and a uniform electric field was generated (HSP, 50 pulses of 20 μs, at a frequency of 10 Hz; LLP, one pulse of 100 ms). The chamber was placed onto the stage of an inverted fluorescence microscope (Ti2-U, Nikon) to visualize 100 μM PI and 1× QDs-647 electrotransfer. For each electrotransfer experiment of siRNAs, MDA-MB-231-GFP and MDA-MB-231-Luci cells were suspended at 1 × 10^6^ cells/ml in Opti-MEM (Thermo, USA) and electroporated with 13 nM siRNA. After incubation for 10 min at 37 °C, cells were transferred onto 6-well plates and the volume increased to 4 ml/well. Then, cells were incubated at 37 °C for 48 h before being analyzed by a fluorescence microscope to determine the percentage of GFP-expressing cells or luciferase-expressing cells and their associated fluorescence intensity. The knockdown efficiency can be calculated as follows:

$$\begin{array}{c} \text{Knockdown efficiency}\;\left(\%\right)=\left(1-\frac{{\mathrm{FL}}_{\text{targeted transfected}}}{{\mathrm{FL}}_{\text{nontargeted transfected}}\;}\right)*100\%, \end{array}$$ (5)

where ${\mathrm{FL}}_{\text{targeted transfected}}$ is the fluorescence intensity of the group transfected with targeted siRNA and ${\mathrm{FL}}_{\text{nontargeted transfected}}$ is the fluorescence intensity of the group transfected with negative control siRNA.

### Cell viability assay

For instant viability analysis, the cells were stained with calcein-AM and PI purchased from Beyotime (Shanghai, China) recovering for 30 min after exposure to PEF. Cells were resuspended in PBS and analyzed for the fluorescence of calcein-AM and PI by flow cytometry (Beckman Coulter Gallios Flow Cytometer). Cells expressing calcein-AM fluorescence (Ex/Em 495 nm/515 nm) were considered alive, while the ones expressing PI fluorescence (Ex/Em 535 nm/617 nm) were considered dead. For long-time viability analysis, the MTT assay was conducted after exposure to an electric field using the MTT Cell Proliferation and Cytotoxicity Assay Kit (Beyotime, Cat. No. C0009M, Shanghai, China). A total of 5,000 treated cells were seeded into each well of a 96-well plate and cultured in 100 μl of medium for 24 h. Then, 10 μl of MTT solution was added into the well. The plate was kept for another 4 h in the incubator. After incubation, 100 μl of formazan dissolution solution was added to each well, followed by further incubation at 37 °C for approximately 3 h until the formazan crystals were fully dissolved.

### Microscopy

Confocal microscopy was used to follow the delivery of PI, QDs, and siRNA. These molecules were detected using a Nikon Ti2-U inverted confocal laser scanning microscope with lasers of wavelengths 561, 647, and 488 nm, respectively. Laser power and photomultiplier settings were identical for all samples so that the results were comparable. All fluorescent images for time-lapse imaging within one sequence were displayed at the same intensity scale. The laser scan was unidirectional and perpendicular to the electric field direction in order to remove temporal delay during image acquisition. To further characterize the electroporation in the cell membrane surface morphology, a GeminiSEM 500 scanning electron microscope (ZEISS, Germany) was used with the following parameters: work distance = 4 mm, accelerating voltage = 1 kV, and magnification = ×20,000. For sample preparation, cells were seeded at 1 × 10^5^/ml on a monocrystalline silicon chip (1 cm × 1 cm) and cultured in 24-well culture dishes with medium for 2 d before electric stimulus [93]. Then, cells were fixed immediately or at different time points (5, 10, 15, and 30 min for recovery) post-electroporation and kept overnight at 4 °C. After dehydration, samples were placed on an aluminum sample dish and coated with 2 nm of gold for optimal imaging.

### In vivo electroporation

Male BALB/c nude mice were from Vital River (Shanghai, China) and were subjected to an adaptation period of at least 10 d before experiments. Mice were maintained at constant room temperature with a 12-h light cycle in a conventional animal colony. The mice were 6 to 8 weeks old at the beginning of the experiments and weighed 18 to 22 g (Table S3).

Procedures were performed with approved protocols (Fig. S8). The cell-derived xenograft model was developed by an intradermal injection of 1 × 10^7^ MDA-MB-231-Luci cells in the left flank of nude mice. The subcutaneous tumors were allowed to grow for 1 week with a final volume of approximately 100 mm^3^ (Table S3). Then, 2 μg of the desired siRNA in 1× PBS was injected at multiple points into the target area using a 23-gauge needle. Immediately following injection, tweezer-type electrodes were used to gently cover the target area, and PEP electric pulses were applied by using the self-built device (Fig. S9). The mice were anesthetized and observed via the IVIS in vivo imaging system, IVIS Lumina Series III (PerkinElmer, USA), at 0 and 2 d after various treatments.

### Statistical analysis

The number of biological and technical replicates and the number of samples are indicated in each figure caption. Data are presented as mean ± standard deviation or standard error of the mean. Statistical analysis was performed by 2-tailed Student *t* tests between 2 groups or by one-way analysis of variance among 3 or more groups using GraphPad Prism 8.0 (GraphPad Software Inc., USA). *P < 0.05, **P < 0.01, ***P < 0.001, and ****P < 0.0001 were considered statistically significant. Every experiment had at least 3 biological and technical parallel replicates.

## Ethical Approval

All animal experiments were carried out in accordance with the Principles of Laboratory Animal Care and approved by the Animal Committee of the Department of Laboratory Science, Fudan University, China (assigned approval number: 202301011Z).

## Data Availability

The data that support the findings of this study are available from the corresponding authors upon reasonable request.

## Supplementary Materials

Supplementary 1 Supplementary Method Figs. S1 to S12 Tables S1 to S3

## References

[1] Stewart MP, Langer R, Jensen KF. Intracellular delivery by membrane disruption: Mechanisms, strategies, and concepts. Chem Rev. 2018;118(16):7409–7531. 30052023 10.1021/acs.chemrev.7b00678 PMC6763210

[2] Wang LL-W, Gao Y, Feng Z, Mooney DJ, Mitragotri S. Designing drug delivery systems for cell therapy. Nat Rev Bioeng. 2024;2:944–959.

[3] Silvestrini AVP, Morais MF, Debiasi BW, Praça FG, Bentley MVLB. Nanotechnology strategies to address challenges in topical and cellular delivery of siRNAs in skin disease therapy. Adv Drug Deliv Rev. 2024;207: Article 115198. 38341146 10.1016/j.addr.2024.115198

[4] Setten RL, Rossi JJ, Han S-P. The current state and future directions of RNAi-based therapeutics. Nat Rev Drug Discov. 2019;18(18):421–446. 30846871 10.1038/s41573-019-0017-4

[5] Ali Zaidi SS, Fatima F, Ali Zaidi SA, Zhou D, Deng W, Liu S. Engineering siRNA therapeutics: Challenges and strategies. J Nanobiotechnol. 2023;21(1):381. 10.1186/s12951-023-02147-z PMC10583313 37848888

[6] Jadhav V, Vaishnaw A, Fitzgerald K, Maier MA. RNA interference in the era of nucleic acid therapeutics. Nat Biotechnol. 2024;42(3):394–405. 38409587 10.1038/s41587-023-02105-y

[7] Qiu C, Xia F, Zhang J, Shi Q, Meng Y, Wang C, Pang H, Gu L, Xu C, Guo Q, et al. Advanced strategies for overcoming endosomal/lysosomal barrier in nanodrug delivery. Research. 2023;6:0148. 37250954 10.34133/research.0148 PMC10208951

[8] Stewart MP, Sharei A, Ding X, Sahay G, Langer R, Jensen KF. *In vitro* and *ex vivo* strategies for intracellular delivery. Nature. 2016;538(7624):183–192. 27734871 10.1038/nature19764

[9] Li Z, Wang Y, Gu Z, Hu Q. Engineering cells for therapy and diagnosis. Nat Rev Bioeng. 2024;2(9):770–784.

[10] Chen Y, Tang S, Cai F, Wan Y. Strategies for small extracellular vesicle-based cancer immunotherapy. Research. 2024;7:0421. 39040921 10.34133/research.0421 PMC11260559

[11] Moazzam M, Zhang M, Hussain A, Yu X, Huang J, Huang Y. The landscape of nanoparticle-based siRNA delivery and therapeutic development. Mol Ther. 2024;32(2):284–312. 38204162 10.1016/j.ymthe.2024.01.005 PMC10861989

[12] Morshedi Rad D, Alsadat Rad M, Razavi Bazaz S, Kashaninejad N, Jin D, Ebrahimi Warkiani M. A comprehensive review on intracellular delivery. Adv Mater. 2021;33(13):2005363. 10.1002/adma.202005363 33594744

[13] Chakrabarty P, Illath K, Kar S, Nagai M, Santra TS. J Control Release. 2023;353:1084–1095. 36538949 10.1016/j.jconrel.2022.12.038

[14] Liu J, Jiang J, He M, Chen J, Huang S, Liu Z, Yao C, Chen H-J, Xie X, Wang J. Nanopore electroporation device for DNA transfection into various spreading and nonadherent cell types. ACS Appl Mater Interfaces. 2023;15(43):50015–50033. 37853502 10.1021/acsami.3c10939

[15] Boukany PE, Morss A, Liao W-C, Henslee B, Jung H, Zhang X, Yu B, Wang X, Wu Y, Li L, et al. Nanochannel electroporation delivers precise amounts of biomolecules into living cells. Nat Nanotechnol. 2011;6(11):747–754. 22002097 10.1038/nnano.2011.164

[16] Kotnik T, Rems L, Tarek M, Miklavčič D. Membrane electroporation and electropermeabilization: Mechanisms and models. Annu Rev Biophys. 2019;48:63–91. 30786231 10.1146/annurev-biophys-052118-115451

[17] Šatkauskas S, Batiuškaitė D, Šalomskaitė-Davalgienė S, Venslauskas MS. Effectiveness of tumor electrochemotherapy as a function of electric pulse strength and duration. Bioelectrochemistry. 2005;65(2):105–111. 15713560 10.1016/j.bioelechem.2004.08.003

[18] Gehl J. Electroporation: Theory and methods, perspectives for drug delivery, gene therapy and research. Acta Physiol Scand. 2003;177(4):437–447. 12648161 10.1046/j.1365-201X.2003.01093.x

[19] Sachdev S, Potočnik T, Rems L, Miklavčič D. Revisiting the role of pulsed electric fields in overcoming the barriers to *in vivo* gene electrotransfer. Bioelectrochemistry. 2022;144: Article 107994. 34930678 10.1016/j.bioelechem.2021.107994

[20] Smith KC, Son RS, Gowrishankar TR, Weaver JC. Emergence of a large pore subpopulation during electroporating pulses. Bioelectrochemistry. 2014;100:3–10. 24290730 10.1016/j.bioelechem.2013.10.009

[21] Weaver JC, Smith KC, Esser AT, Son RS, Gowrishankar TR. A brief overview of electroporation pulse strength–duration space: A region where additional intracellular effects are expected. Bioelectrochemistry. 2012;87:236–243. 22475953 10.1016/j.bioelechem.2012.02.007 PMC3423488

[22] Yarmush ML, Golberg A, Serša G, Kotnik T, Miklavčič D. Electroporation-based technologies for medicine: Principles, applications, and challenges. Annu Rev Biomed Eng. 2014;16:295–320. 24905876 10.1146/annurev-bioeng-071813-104622

[23] Batista Napotnik T, Polajžer T, Miklavčič D. Cell death due to electroporation—A review. Bioelectrochemistry. 2021;141: Article 107871. 34147013 10.1016/j.bioelechem.2021.107871

[24] Kumar P, Nagarajan A, Uchil PD. Electroporation. Cold Spring Harb Protoc. 2019;7: Article pdb.top096271. 10.1101/pdb.top096271 31262965

[25] André FM, Gehl J, Sersa G, Préat V, Hojman P, Eriksen J, Golzio M, Cemazar M, Pavselj N, Rols MP, et al. Efficiency of high- and low-voltage pulse combinations for gene electrotransfer in muscle, liver, tumor, and skin. Hum Gene Ther. 2008;19(11):1261–1272. 19866490 10.1089/hum.2008.060

[26] Stroh T, Erben U, Kühl AA, Zeitz M, Siegmund B. Combined pulse electroporation—A novel strategy for highly efficient transfection of human and mouse cells. PLOS ONE. 2010;5(3): Article e9488. 20209146 10.1371/journal.pone.0009488 PMC2830457

[27] Pathak N, Patino CA, Ramani N, Mukherjee P, Samanta D, Ebrahimi SB, Mirkin CA, Espinosa HD. Cellular delivery of large functional proteins and protein–nucleic acid constructs via localized electroporation. Nano Lett. 2023;23(8):3653–3660. 36848135 10.1021/acs.nanolett.2c04374 PMC10433461

[28] Ding X, Stewart MP, Sharei A, Weaver JC, Langer RS, Jensen KF. High-throughput nuclear delivery and rapid expression of DNA via mechanical and electrical cell-membrane disruption. Nat Biomed Eng. 2017;1(3):0039. 28932622 10.1038/s41551-017-0039 PMC5602535

[29] Pavlin M, Kandušer M. New insights into the mechanisms of gene electrotransfer—Experimental and theoretical analysis. Sci Rep. 2015;5(1):9132. 25778848 10.1038/srep09132 PMC5390920

[30] Wang Y, Qian K, Liu X, Yang Q, Lei Y, Zhu L, Yao C, Zhou Q, Liu H, Dong S. Synergistic bipolar irreversible electroporation for tumor ablation without muscle contraction. IEEE Trans Biomed Eng. 2024;71(12):3505–3514. 39028604 10.1109/TBME.2024.3431013

[31] Emon S, Amin A, Hossain M, Saha S, Asaduzzaman M, Hossen ML, Karal MAS, Takaba H, Alam MK. Optimizing electroporation via pulse modulation: a molecular dynamics study. Eur Biophys J. 2025;54(7):477–490. 40841485 10.1007/s00249-025-01793-5

[32] Demiryurek Y, Nickaeen M, Zheng M, Yu M, Zahn JD, Shreiber DI, Lin H, Shan JW. Transport, resealing, and re-poration dynamics of two-pulse electroporation-mediated molecular delivery. Biochim Biophys Acta Biomembr. 2015;1848(8):1706–1714. 10.1016/j.bbamem.2015.04.007 25911207

[33] Palepšienė R, Muralidharan A, Maciulevičius M, Ruzgys P, Chopra S, Boukany PE, Šatkauskas S. New insights into the mechanism of electrotransfer of small nucleic acids. Bioelectrochemistry. 2024;158: Article 108696. 38583283 10.1016/j.bioelechem.2024.108696

[34] Rols M-P, Teissié J. Electropermeabilization of mammalian cells to macromolecules: Control by pulse duration. Biophys J. 1998;75(3):1415. 9726943 10.1016/S0006-3495(98)74060-3 PMC1299816

[35] Weaver JC, Vernier PT. Pore lifetimes in cell electroporation: Complex dark pores? arXiv. 2017. 10.48550/arXiv.1708.07478

[36] Müller WA, Sarkis JR, Marczak LDF, Muniz AR. Molecular dynamics insights on temperature and pressure effects on electroporation. Biochim Biophys Acta Biomembr. 2022;1864(12): Article 184049. 36113558 10.1016/j.bbamem.2022.184049

[37] Hub JS. Joint reaction coordinate for computing the free-energy landscape of pore nucleation and pore expansion in lipid membranes. J Chem Theory Comput. 2021;17(2):1229–1239. 33427469 10.1021/acs.jctc.0c01134

[38] Kasparyan G, Hub JS. Molecular simulations reveal the free energy landscape and transition state of membrane electroporation. Phys Rev Lett. 2024;132(14): Article 148401. 38640376 10.1103/PhysRevLett.132.148401

[39] Rems L, Tang X, Zhao F, Pérez-Conesa S, Testa I, Delemotte L. Identification of electroporation sites in the complex lipid organization of the plasma membrane. eLife. 2022;11: Article e74773. 35195069 10.7554/eLife.74773 PMC8912918

[40] Guo F, Xiang J, Zhuo Y, Pei K. Molecular dynamics study of protein-mediated electroporation of Kv channels induced by nsPEFs: Advantages of bipolar pulses. Biomacromolecules. 2025;26:1002. 39808923 10.1021/acs.biomac.4c01321

[41] Sözer EB, Haldar S, Blank PS, Castellani F, Vernier PT, Zimmerberg J. Dye transport through bilayers agrees with lipid electropore molecular dynamics. Biophys J. 2020;119(9):1724–1734. 33096018 10.1016/j.bpj.2020.09.028 PMC7677249

[42] Barnett A, Weaver JC. Electroporation: A unified, quantitative theory of reversible electrical breakdown and mechanical rupture in artificial planar bilayer membranes. J Electroanal Chem Interfacial Electrochem. 1991;320(2):163–182.

[43] Glaser RW, Leikin SL, Chernomordik LV, Pastushenko VF, Sokirko AI. Reversible electrical breakdown of lipid bilayers: Formation and evolution of pores. Biochim Biophys Acta Biomembr. 1988;940:275–287. 10.1016/0005-2736(88)90202-7 2453213

[44] Lafarge EJ, Muller P, Schroder AP, Zaitseva E, Behrends JC, Marques CM. Activation energy for pore opening in lipid membranes under an electric field. Proc Natl Acad Sci USA. 2023;120(11): Article e2213112120. 36881617 10.1073/pnas.2213112120 PMC10089165

[45] Behera N, Thaokar RM. Numerical modeling of giant pore formation in vesicles under msPEF-induced electroporation: Role of charging time and waveform. Bioelectrochemistry. 2025;164: Article 108926. 39929135 10.1016/j.bioelechem.2025.108926

[46] Zaharoff DA, Henshaw JW, Mossop B, Yuan F. Mechanistic analysis of electroporation-induced cellular uptake of macromolecules. Exp Biol Med. 2008;233(1):94–105. 10.3181/0704-RM-113 PMC2782745 18156311

[47] Venslauskas MS, Šatkauskas S. Mechanisms of transfer of bioactive molecules through the cell membrane by electroporation. Eur Biophys J. 2015;44(5):277–289. 25939984 10.1007/s00249-015-1025-x

[48] Gabriel B, Teissié J. Direct observation in the millisecond time range of fluorescent molecule asymmetrical interaction with the electropermeabilized cell membrane. Biophys J. 1997;73:2630–2637. 9370457 10.1016/S0006-3495(97)78292-4 PMC1181165

[49] Rems L, Viano M, Kasimova MA, Miklavčič D, Tarek M. The contribution of lipid peroxidation to membrane permeability in electropermeabilization: A molecular dynamics study. Bioelectrochemistry. 2019;125:46–57. 30265863 10.1016/j.bioelechem.2018.07.018

[50] Wiczew D, Szulc N, Tarek M. Molecular dynamics simulations of the effects of lipid oxidation on the permeability of cell membranes. Bioelectrochemistry. 2021;141: Article 107869. 34119820 10.1016/j.bioelechem.2021.107869

[51] Qian K, Wang Y, Lei Y, Yang Q, Yao C. An experimental and theoretical study on cell swelling for osmotic imbalance induced by electroporation. Bioelectrochemistry. 2024;157: Article 108637. 38215652 10.1016/j.bioelechem.2023.108637

[52] Lira RB, Leomil FSC, Melo RJ, Riske KA, Dimova R. To close or to collapse: The role of charges on membrane stability upon pore formation. Adv Sci. 2021;8(11):2004068. 10.1002/advs.202004068 PMC8188222 34105299

[53] Luz JC, Antunes F, Clavijo-Salomon MA, Signori E, Tessarollo NG, Strauss BE. Clinical applications and immunological aspects of electroporation-based therapies. Vaccine. 2021;9(7): Article 727. 10.3390/vaccines9070727 PMC8310106 34358144

[54] Matsuda T, Cepko CL. Electroporation and RNA interference in the rodent retina *in vivo* and *in vitro*. Proc Natl Acad Sci USA. 2004;101(1):16–22. 14603031 10.1073/pnas.2235688100 PMC314130

[55] Bureau MF, Gehl J, Deleuze V, Mir LM, Scherman D. Importance of association between permeabilization and electrophoretic forces for intramuscular DNA electrotransfer. Biochim Biophys Acta Gen Subj. 2000;1474(3):353–359. 10.1016/s0304-4165(00)00028-3 10779687

[56] Pavšelj N, Préat V. DNA electrotransfer into the skin using a combination of one high- and one low-voltage pulse. J Control Release. 2005;106(3):407–415. 15982778 10.1016/j.jconrel.2005.05.003

[57] Pan Y, Zhang Y, Shi X, Li D, Xu X, Xiao B, Piao Y, Xiang J, Shao S, Ho FC-Y, et al. Electrical stimulation induces anti-tumor immunomodulation via a flexible microneedle-array-integrated interdigital electrode. Sci Bull. 2023;68(22):2779–2792. 10.1016/j.scib.2023.10.004 37863773

[58] Bernelin-Cottet C, Urien C, McCaffrey J, Collins D, Donadei A, McDaid D, Jakob V, Barnier-Quer C, Collin N, Bouguyon E, et al. Electroporation of a nanoparticle-associated DNA vaccine induces higher inflammation and immunity compared to its delivery with microneedle patches in pigs. J Control Release. 2019;308:14–28. 31265882 10.1016/j.jconrel.2019.06.041

[59] Campelo SN, Huang PH, Buie CR, Davalos RV. Recent advancements in electroporation technologies: From bench to clinic. Annu Rev Biomed Eng. 2023;25:77–100. 36854260 10.1146/annurev-bioeng-110220-023800 PMC11633374

[60] Aldawood FK, Andar A, Desai S. A comprehensive review of microneedles: Types, materials, processes, characterizations and applications. Polymers. 2021;13(16):2815. 34451353 10.3390/polym13162815 PMC8400269

[61] Chang DC, Reese TS. Changes in membrane structure induced by electroporation as revealed by rapid-freezing electron microscopy. Biophys J. 1990;58(1):1–12. 2383626 10.1016/S0006-3495(90)82348-1 PMC1280935

[62] Sengel JT, Wallace MI. Imaging the dynamics of individual electropores. Proc Natl Acad Sci USA. 2016;113(19):5281. 27114528 10.1073/pnas.1517437113 PMC4868429

[63] Silkunas M, Silkuniene G, Pakhomov AG. Real-time imaging of individual electropores proves their longevity in cells. Biochem Biophys Res Commun. 2024;695: Article 149408. 38157631 10.1016/j.bbrc.2023.149408 PMC10842338

[64] Wang Y, Lu Y-W, Liu H. Sulfonated hydrogel electrolyte enables dendrite-free zinc-ion batteries. Chem Eng J. 2024;479: Article 147801.

[65] Liu Y, Fan Z, Xiang X-W, Tao X, Xia X, Shi Q, Lu Y, Lu J, Gu H, Liu Y-J, et al. Engineering of multivalent membrane-anchored DNA frameworks for precise profiling of variable membrane permeability during reversible electroporation. Small Methods. 2023;8(7): Article 2301198. 10.1002/smtd.202301198 38152955

[66] Akimov SA, Volynsky PE, Galimzyanov TR, Kuzmin PI, Pavlov KV, Batishchev OV. Pore formation in lipid membrane II: Energy landscape under external stress. Sci Rep. 2017;7(1):12509. 28970526 10.1038/s41598-017-12749-x PMC5624950

[67] Novickij V, Rembiałkowska N, Szlasa W, Kulbacka J. Does the shape of the electric pulse matter in electroporation? Front Oncol. 2022;12: Article 958128. 36185267 10.3389/fonc.2022.958128 PMC9518825

[68] Baker C, Milestone W, Garner AL, Joshi RP. Selective electroporation of tumor cells under AC radiofrequency stimulation: A numerical study. IEEE Trans Biomed Eng. 2024;71(1):114–121. 10.1109/TBME.2023.3293278 37418405

[69] Hemmerle A, Fragneto G, Daillant J, Charitat T. Reduction in tension and stiffening of lipid membranes in an electric field revealed by x-ray scattering. Phys Rev Lett. 2016;116(22): Article 228101. 27314739 10.1103/PhysRevLett.116.228101

[70] Gandhi SA, Kelly CV. Membrane asymmetry enhances nanotube formation and limits pore resealing after electroporation. Biophys J. 2022;121(17):3173–3174. 35973422 10.1016/j.bpj.2022.07.037 PMC9463694

[71] Wadud MA, Karal MAS, Moniruzzaman M, Rashid MMO. Effects of membrane potentials on the electroporation of giant unilamellar vesicles. PLOS ONE. 2023;18(9): Article e0291496. 37699026 10.1371/journal.pone.0291496 PMC10497157

[72] Zhelev DV, Needham D. Tension-stabilized pores in giant vesicles: Determination of pore size and pore line tension. Biochim Biophys Acta. 1993;1147(1):89–104. 8466935 10.1016/0005-2736(93)90319-u

[73] Liu H, Xiang X, Tao X, Zhao H, Qiu J, Liu K. An electroporation-mediated intracellular delivery model with coupled pore currents and solute transport. IEEE Trans Dielectr Electr Insul. 2024;31(4):1971–1980.

[74] Goldberg E, Soba A, Gandía D, Fernández ML, Suárez C. Coupled mathematical modeling of cisplatin electroporation. Bioelectrochemistry. 2021;140: Article 107788. 33838515 10.1016/j.bioelechem.2021.107788

[75] Li J, Lin H. Numerical simulation of molecular uptake via electroporation. Bioelectrochemistry. 2011;82(1):10–21. 21621484 10.1016/j.bioelechem.2011.04.006

[76] Guo F, Qian K, Zhang L, Liu X, Peng H. Multiphysics modelling of electroporation under uni- or bipolar nanosecond pulse sequences. Bioelectrochemistry. 2021;141: Article 107878. 34198114 10.1016/j.bioelechem.2021.107878

[77] Pucihar G, Miklavcic D, Kotnik T. A time-dependent numerical model of transmembrane voltage inducement and electroporation of irregularly shaped cells. IEEE Trans Biomed Eng. 2009;56(5):1491–1501. 19203876 10.1109/TBME.2009.2014244

[78] Guo F, Zou S, Wei X, Liu L. The impact of cell electrodeformation on dynamic electroporation: A multiphysics coupled model. Electrochim Acta. 2025;522: Article 145961.

[79] Scuderi M, Dermol-Černe J, Amaral da Silva C, Muralidharan A, Boukany PE, Rems L. Models of electroporation and the associated transmembrane molecular transport should be revisited. Bioelectrochemistry. 2022;147: Article 108216. 35932533 10.1016/j.bioelechem.2022.108216

[80] Zhang M, Xu D, Fang J, Li H, Li Y, Liu C, Cao N, Hu N. A dynamic and quantitative biosensing assessment for electroporated membrane evolution of cardiomyocytes. Biosens Bioelectron. 2022;202: Article 114016. 35091372 10.1016/j.bios.2022.114016

[81] Yin H, Kanasty RL, Eltoukhy AA, Vegas AJ, Dorkin JR, Anderson DG. Non-viral vectors for gene-based therapy. Nat Rev Genet. 2014;15(8):541–555. 25022906 10.1038/nrg3763

[82] Bashor CJ, Hilton IB, Bandukwala H, Smith DM, Veiseh O. Engineering the next generation of cell-based therapeutics. Nat Rev Drug Discov. 2022;21(9):655–675. 35637318 10.1038/s41573-022-00476-6 PMC9149674

[83] Antov Y, Barbul A, Mantsur H, Korenstein R. Electroendocytosis: Exposure of cells to pulsed low electric fields enhances adsorption and uptake of macromolecules. Biophys J. 2005;88(3):2206–2223. 15556977 10.1529/biophysj.104.051268 PMC1305271

[84] Rosazza C, Deschout H, Buntz A, Braeckmans K, Rols M-P, Zumbusch A. Endocytosis and endosomal trafficking of DNA after gene electrotransfer in vitro. Mol Ther Nucleic Acids. 2016;5(2): Article e286. 26859199 10.1038/mtna.2015.59 PMC4884790

[85] Bulysheva A, Heller L, Francis M, Varghese F, Boye C, Heller R. Monopolar gene electrotransfer enhances plasmid DNA delivery to skin. Bioelectrochemistry. 2021;140: Article 107814. 33962133 10.1016/j.bioelechem.2021.107814

[86] Abbasi M, Heath B. Iontophoresis and electroporation-assisted microneedles: Advancements and therapeutic potentials in transdermal drug delivery. Drug Deliv Transl Res. 2025;15(6):1962–1984. 39433696 10.1007/s13346-024-01722-7 PMC12037666

[87] Liu H, Tao X, Xiang X, Zhao H, Qiu J, Liu K. An electroporation-mediated intracellular delivery model with coupled pore currents and solute transport. IEEE Trans Dielectr Electr Insul. 2023;31(4):1971–1980.

[88] Rocha LL, Silva JF, Redondo LM. Multilevel high-voltage pulse generation based on a new modular solid-state switch. IEEE Trans Plasma Sci. 2014;42(10):2956–2961.

[89] Elgenedy MA, Massoud AM, Ahmed S, Williams BW, McDonald JR. A modular multilevel voltage-boosting Marx pulse-waveform generator for electroporation applications. IEEE Trans Power Electron. 2019;34(11):10575.

[90] Wang K, Xie S, Ren Y, Xia H, Zhang X, He J. Bioinformatics analysis of miRNAs germacrone protection on diabetic nephropathy. Oncol Rep. 2012;27(1): Article 1981. 39738220 10.1038/s41598-024-81944-4 PMC11685625

[91] Zhang L-J, Xia L, Xie H-Y, Zhang Z-L, Pang D-W. Quantum dot based biotracking and biodetection. Anal Chem. 2019;91(1):532–547. 30532950 10.1021/acs.analchem.8b04721

[92] Li S-D, Chono S, Huang L. Efficient oncogene silencing and metastasis inhibition via systemic delivery of siRNA. Mol Ther. 2008;16(5):942–946. 18388916 10.1038/mt.2008.51 PMC2778045

[93] Pan H-A, Hung Y-C, Su C-W, Tai S-M, Chen C-H, Ko F-H, Steve Huang G. A nanodot array modulates cell adhesion and induces an apoptosis-like abnormality in NIH-3T3 cells. Nanoscale Res Lett. 2009;4(8):903–912. 20596320 10.1007/s11671-009-9333-7 PMC2894250

