# Supplementary material for: Regulation of Pore Evolution via Progressive Electroporation Enhanced Intracellular Molecule Transport
Source: Research (Wash D C). 2026 Jan 23;9:1095. doi: 10.34133/research.1095 (PMC13274628; doi:10.34133/research.1095)
Supplement: Supplementary 1 — Supplementary Method Figs. S1 to S12 Tables S1 to S3 [file research.1095.f1.docx]

**Supplementary Materials**

**for**

**Regulation of Pore Evolution via Progressive Electroporation Enhanced Intracellular Molecule Transport**

*Xiao-Nan Tao^a,1^, Xiao-Wei Xiang^b,1^, Hao-Tian Liu^b^, Cai-Hui Zhu^a^,* *Jing Liu^b^, Ya-Jun Wang^c^, Wei Liu^c^, Yu-Chen Chen^c^, Yu-Lian Zeng^d^, Sai-Xi Yu^c^, Jian Qiu^a^, Guangyin Jing^e^, Hui Zhao^a^, Qi-Hong Huang^f,*^, Yan-Jun Liu^c,*^, Ke-Fu Liu^a,*^*

*^a^* School of Information Science and Technology, Fudan University, Shanghai, 200433, China.

*^b^* Academy for Engineering & Technology, Fudan University, Shanghai, 200433, China.

*^c^* Shanghai Key Laboratory of Medical Epigenetics, International Co-laboratory of Medical Epigenetics and Metabolism (Ministry of Science and Technology), Institutes of Biomedical Sciences, Fudan University, Shanghai, 200032, China.

*^d^* Ruijin Hospital, Shanghai Jiao Tong University School of Medicine, Shanghai 200025, China

*^e^* School of Physics, State Key Laboratory of Photon Technology in Western China Energy, Northwest University, Xi'an, 710127, China.

*^f^* Zhongshan Hospital Institute of Clinical Science, Shanghai Medical School, Fudan University, Shanghai, 200032, China.

^1^These authors contribute equally to this work.

*Corresponding Authors.

Email address: [kfliu@fudan.edu.cn](mailto:kfliu@fudan.edu.cn) (K.-F. Liu); orcid.org/0000-0001-8249-2800; [Yanjun_Liu@fudan.edu.cn](mailto:Yanjun_Liu@fudan.edu.cn) (Y.-J. Liu); orcid.org/0000-0001-6535-8431; [qihong_huang@fudan.edu.cn](mailto:qihong_huang@fudan.edu.cn) (Q.-H. Huang); orcid.org/0000-0001-6083-710X

**Contents**

**Fig. S1 Overview of the computational and experimental framework.**

**Table S1 Detailed parameters for different pulse modulation modes.**

**Fig. S2 Effects of total pulse durations and pulse combinations with the same electric energy output on electropermeabilization efficiency and cell survival (A), cell viability at 24 h (B).**

**Fig. S3 Subsequent LLP retards the decrease of transmembrane voltage and interferes the pore contraction.**

**Fig. S4 Free energy distribution dependent on time at 0° and 45° polarization angle.**

**Table S2 Simulation parameters of the cell electroporation model.**

**Fig. S5 Viability analysis.**

**Fig. S6 Lipid peroxidation assay with BODIPY 581/591 C11 under different treatment.**

**Fig. S7 Cell swelling under different electric exposure.**

**Fig. S8 *In vivo* electroporation protocol.**

**Table S3 Tumor volume after 7-day growth.**

**Fig. S9 *In vivo* electroporation experimental device setup.**

**Fig. S10 Changes in pH for electroporated buffer under different treatment.**

**Fig. S11 Regulation of pore resealing dynamics by progressive electroporation facilitated intracellular delivery.**

**Fig. S12 Colocalization analysis of lysosome and FAM-siRNA after two-hour incubation with RNAiMAX reagent or post PEP exposure.**

**Supplementary Figures and Tables**


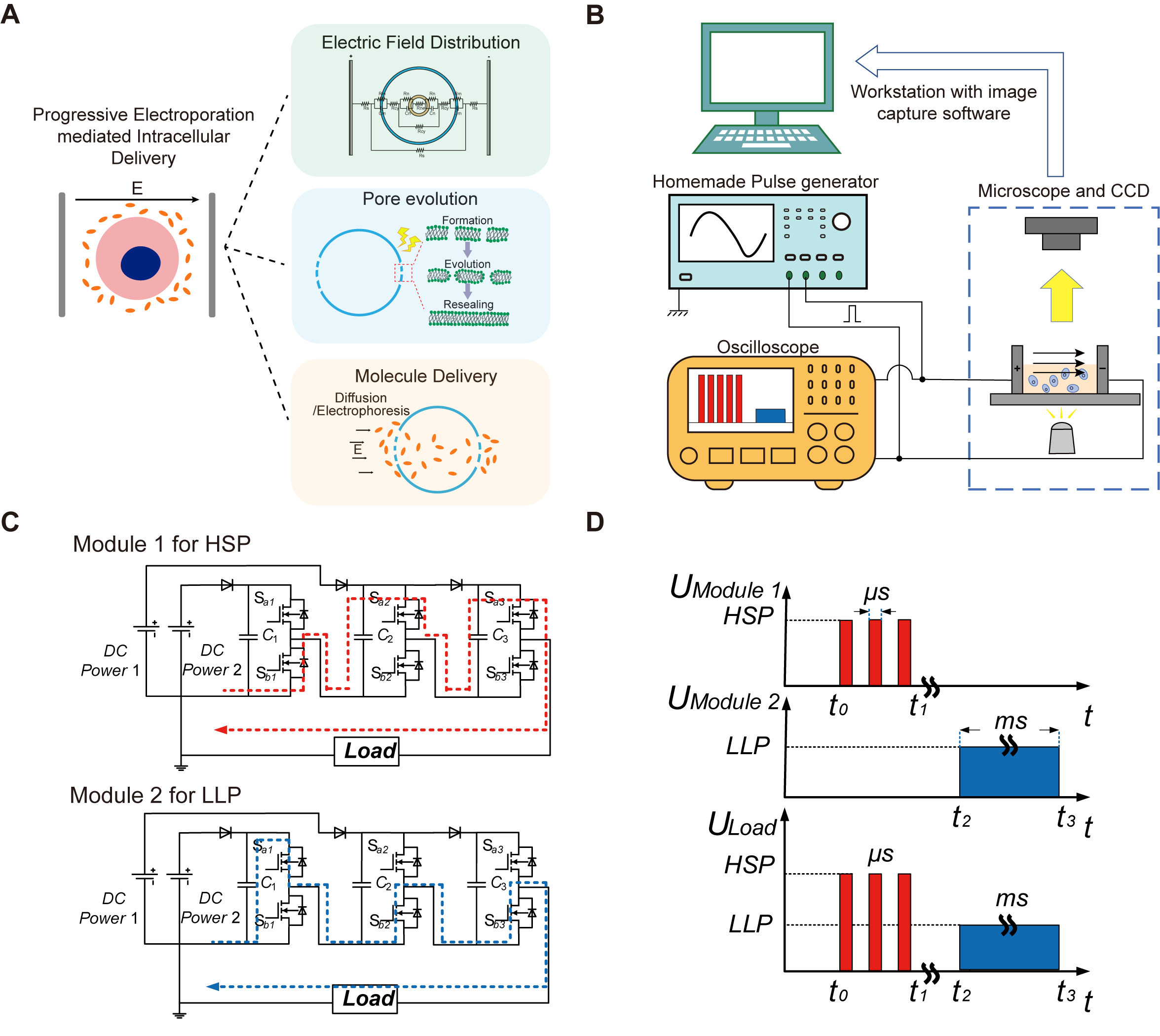


**Fig. S1 Overview of the computational and experimental framework**

(A) Multiphysics field coupled computational framework. (B) Schematic diagram of the experimental setup of the electroporation chamber connected to a pulse generator and an oscilloscope. The optical path is indicated in yellow. (C) Schematic diagram of n-stage solid-state Marx modulators. High-voltage microsecond pulse and low-voltage millisecond pulse topology. (D) A diagram of the controls signals and output pulses. The red column represents HSP, the blue column represents LLP.

**Table S1 Detailed parameters for different pulse modulation modes.**

| **Mode** | **Pulse Modulation** | **Parameters for HSP** | **Parameters for LLP** |
| --- | --- | --- | --- |
| Mode 1 | HSP | 750 V/cm, 20 μs, 10 Hz | - |
| Mode 2 | LLP | - | 75 V/cm, 100 ms |
| Mode 3 | HSP+LLP_1_ | 750 V/cm, 20 μs, 10 Hz | 37.5 V/cm, 100 ms |
| Mode 4 | HSP+LLP_2_ |  | 75 V/cm, 50 ms |
| Mode 5 | HSP+LLP_3_ |  | 75 V/cm, 100 ms |

**
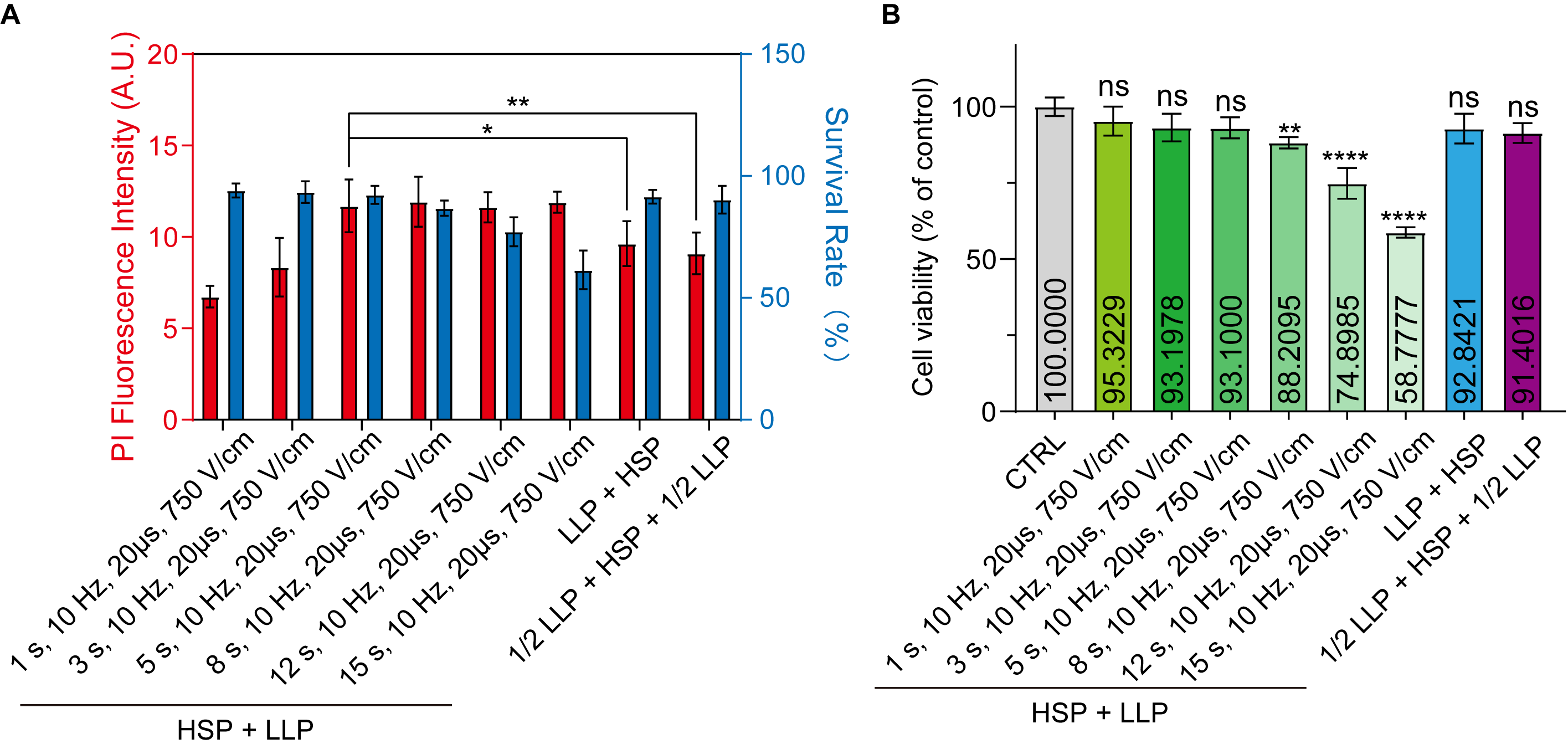
**

**Fig. S2 Effects of total pulse durations and pulse combinations with the same electric energy output on electropermeabilization efficiency and cell survival (A), cell viability at 24 h (B).** LLP used in the experiment is 75 V/cm, 100 ms, 1 pulse. Results are expressed as mean ± standard error of the mean with a 95 % CI (n = 5).


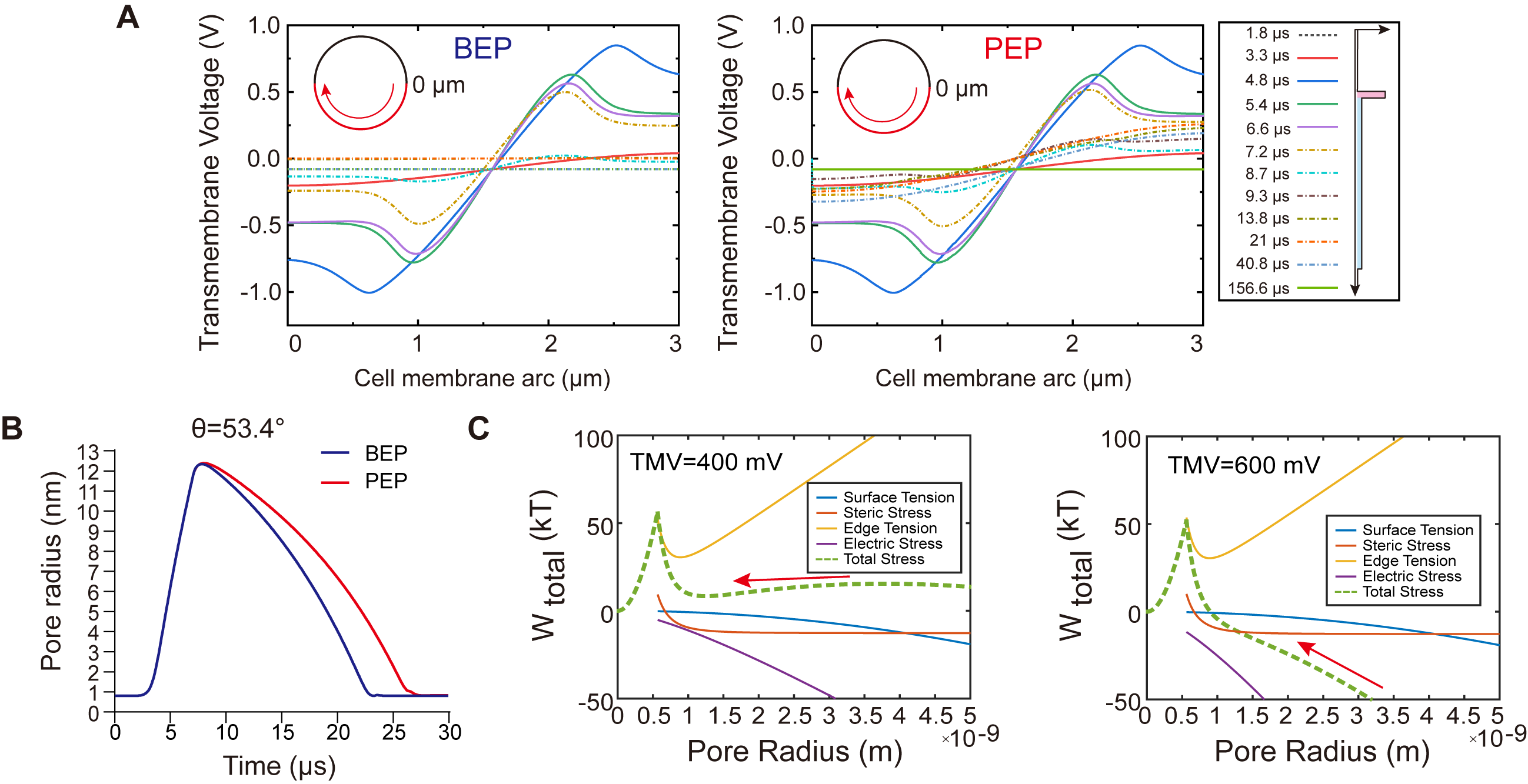


**Fig. S3 Subsequent LLP retards the decrease of transmembrane voltage and interferes the pore contraction.** (A) Transmembrane voltage distribution along the cell membrane with BEP and PEP respectively. (B) Pore evolution at *θ* = 53.4° with the maximum pore radius. (C) Transmembrane voltage reverses the energy variation of pore contraction for extra energy demand. Red arrow indicates the pore energy evolutional gradient with pore contraction.


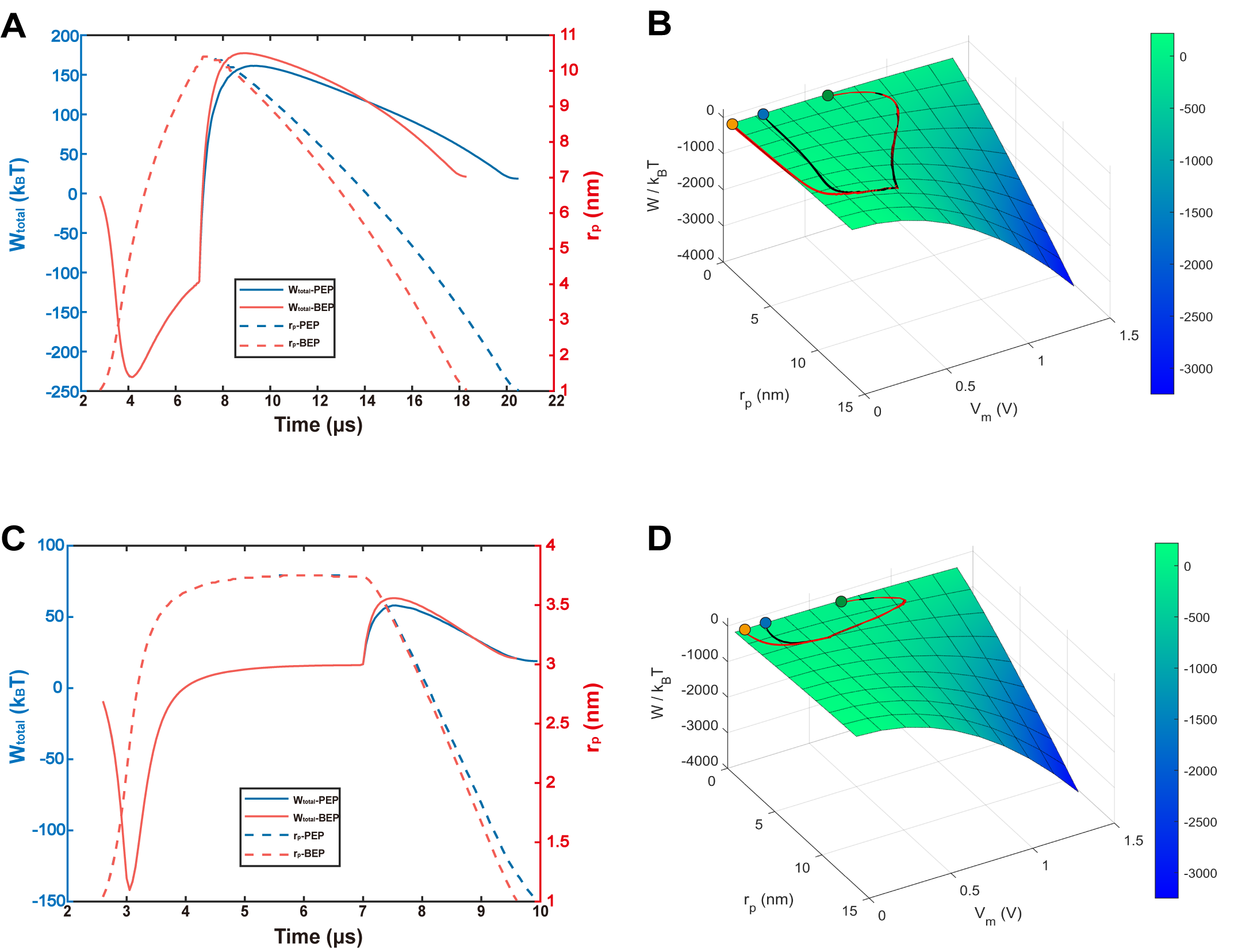


**Fig. S4 Free energy distribution dependent on time at 0° and 45° polarization angle.** (A) and (C), pore energy distribution and pore radius of hydrophilic pores under BEP and PEP. (B) and (D) trajectory of pore evolution in the Wtotal–rp-Vm space. Green circle indicating the starting point, while the orange and blue markers denoting the endpoints of BEP and PEP, respectively.

**Table S2 Simulation parameters of the cell electroporation model**

Parameters for the model were obtained from relevant literature. The important parameters used in the model are listed below.

| **PARAMETER** | **VALUE [unit]** | **REFERENCE** |
| --- | --- | --- |
| $R_{c}$, Cell radius | 10[μm] |  |
| $d_{mem}$, Thickness of the cell membrane | 5[nm] | ^[1]^ |
| $NCR$, Ratio of the nucleus and cytoplasm | 0.02 | ^[2]^ |
| $d_{ne}$, Thickness of the nuclear membrane | 40[nm] | ^[3]^ |
| $\varepsilon_{mem}$, Cell membrane relative permittivity | 8.57 | ^[2, 4]^ |
| $\sigma_{mem0}$, Cell membrane conductivity | 3×10^-7^[S/m] | ^[2, 4]^ |
| $\varepsilon_{ne}$, Nuclear membrane relative permittivity | 28 | ^[2, 4]^ |
| $\sigma_{ne}$, Nuclear membrane conductivity | 6×10^-3^[S/m] | ^[2, 4]^ |
| $\varepsilon_{e}$, Extracellular medium relative permittivity | 80 | ^[2, 4]^ |
| $\sigma_{e}$, Extracellular medium conductivity | 0.2[S/m] | ^[2, 4]^ |
| $\varepsilon_{i}$, Cytoplasm relative permittivity | 154.4 | ^[2, 4]^ |
| $\sigma_{i}$, Cytoplasm conductivity | 0.3[S/m] | ^[2, 4]^ |
| $\varepsilon_{nc}$, Nucleoplasm relative permittivity | 52 | ^[2, 4]^ |
| $\sigma_{nc}$, Nucleoplasm conductivity | 1.35[S/m] | ^[2, 4]^ |
| $\sigma_{p}$, Pore conductivity | 1.3[S/m] | ^[5]^ |
| $\alpha$, Coefficient constant | 1×10^9^[1/(m^2^·s)] | ^[6]^ |
| $\omega_{0}$, Energy barrier within pores | 2.65 | ^[6]^ |
| $q$, Coefficient for pore forming | 2.46 | ^[6]^ |
| $n$, Relative entrance length of pores | 0.15 | ^[6]^ |
| $F$, Faraday constant | 96485[C/mol] |  |
| $R$, Gas constant | 8314[J/(mol·K)] |  |
| $\varepsilon_{0}$, Vacuum permittivity | 8.85×10^-12^[F/m] |  |
| $T$, Ambient temperature | 295[K] |  |
| $N_{0}$, Equilibrium pore density when the transmembrane potential is zero | 1.5×10^9^[1/m^2^] | ^[7]^ |
| $\beta$, Steric repulsion energy | 1.4×10^-19^[J] | ^[8]^ |
| $\gamma$, Edge energy | 1.8×10^-11^[J/m] | ^[8]^ |
| $r_{h}$, Constant of advection velocity | 0.97×10^-9^[m] | ^[8]^ |
| $r_{t}$, Constant of advection velocity | 0.31×10^-9^[m] | ^[9]^ |
| $V_{ep}$, Characteristic voltage of electroporation | 190[mV] | ^[9]^ |
| $D$, Diffusion coefficient of the pore radius | 5×10^-14^[m^2^/s] | ^[1]^ |
| $\sigma^{'}$, Energy of the hydrocarbon-water interface per area | 2×10^-2^[J/m^2^] | ^[7]^ |
| $\sigma_{0}$, Tension of the intact cell membrane | 1×10^-3^[J/m^2^] | ^[4]^ |
| $F_{max}$, Maximum electric force | 0.7×10^-9^[N/V^2^] | ^[6]^ |
| $r_{min}$, Pore radius at minimum energy | 0.76×10^-9^[m] | ^[3]^ |
| $r^{*}$, Minimum radius of hydrophilic pore | 0.51×10^-9^[m] | ^[6]^ |
| $k$, Boltzmann constant | 1.380649×10^-23^ [J/K] |  |


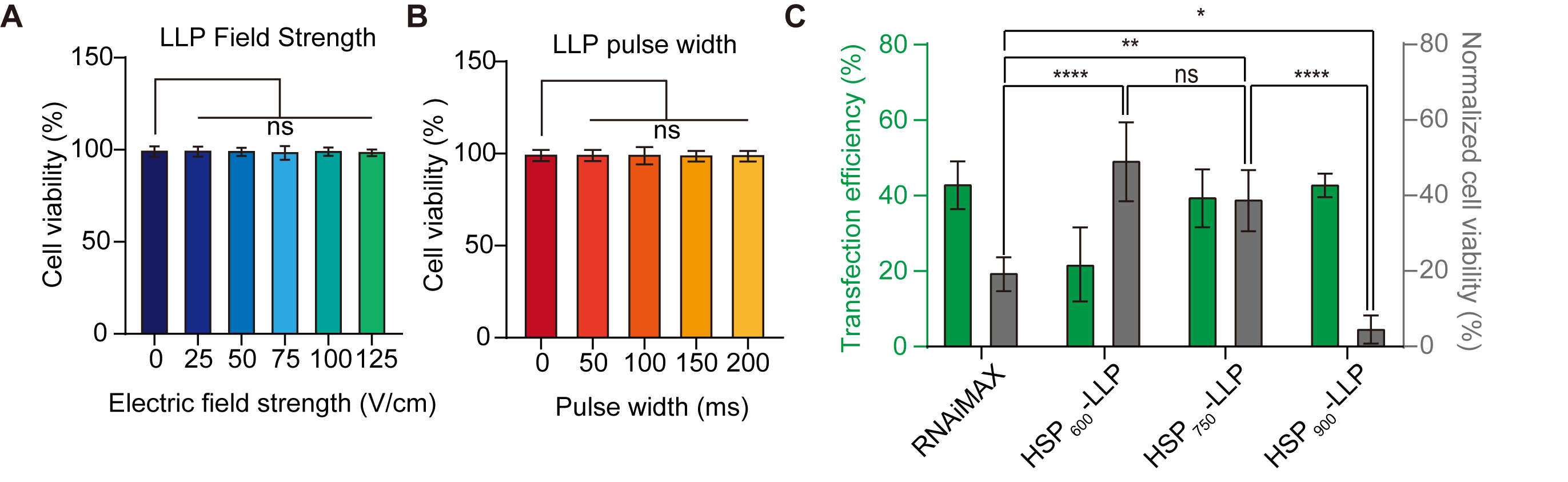


**Fig. S5 Viability analysis.** (A) Effect of different electric field strengths on cell viability immediately after exposure electric field by flow cytometry. (B) Effect of different pulse widths on cell viability immediately after exposure electric field by flow cytometry. (C) Effects of HSP with different field strengths on the siRNA transfection efficiency and MDA-MB-231-GFP cell viability after 48-hour incubation. Quantification of GFP expression after applying the siRNA delivered by the transfection reagent RNAiMAX or electroporation with different HSP field strengths (e.g., HSP_600_ = 600 V/cm, HSP_750_ = 750 V/cm and HSP_900_ = 900 V/cm). Results are expressed as mean ± standard error of the mean with 95% CI. In (C), significance was evaluated by one-way ANOVA for multiple comparisons. ^*^p<0.05, ^**^p<0.01, ^***^p<0.001, ^****^p<0.0001.


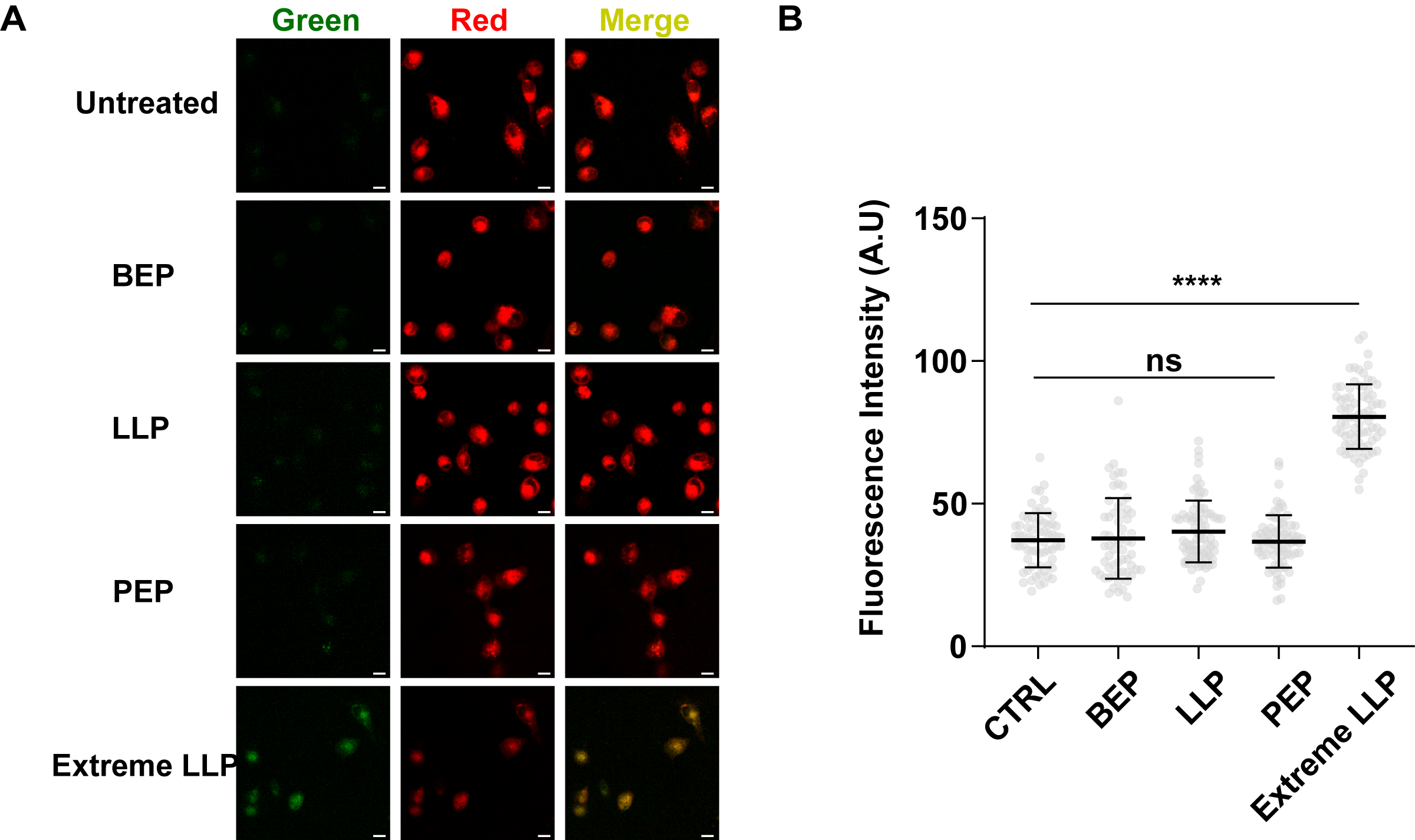


**Fig. S6 Lipid peroxidation assay with BODIPY 581/591 C11 under different treatment.** (A) Representative fluorescent images. Scale bar, 10 μm. (B) Quantification of fluorescence intensity for oxidized lipids. Cells were treated with 125 V/cm, 300 ms, 5 pulses in the Extreme LLP group. Results are expressed as mean ± standard error of the mean with 95% CI. Significance was evaluated by ordinary one-way ANOVA for multiple comparisons, ^****^p<0.001.


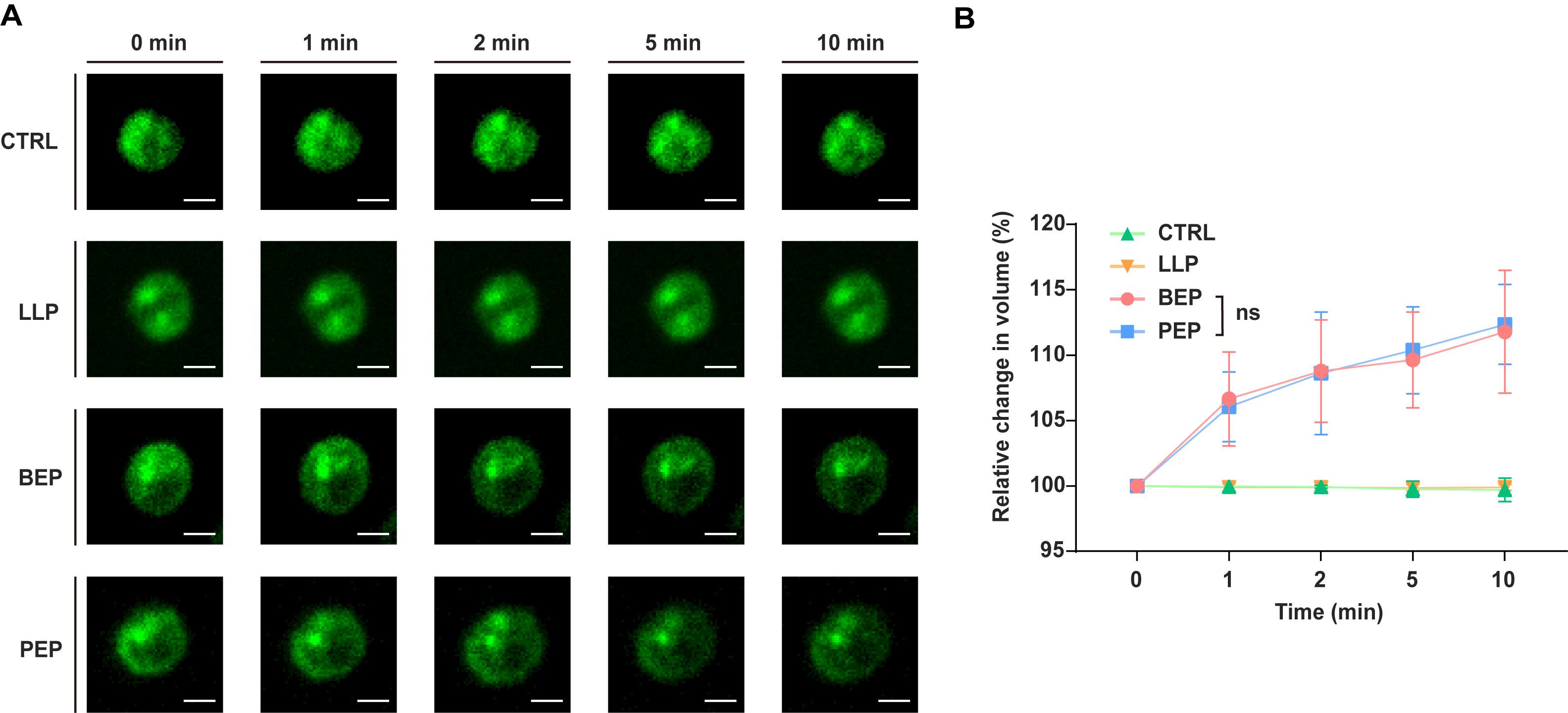


**Fig. S7 Cell swelling under different electric exposure.** (A) representative fluorescent images of cell. Cell membrane was stained with DiO with standard protocol. (B) quantification of relative change in volume. Normalized to the volume at 0 min.


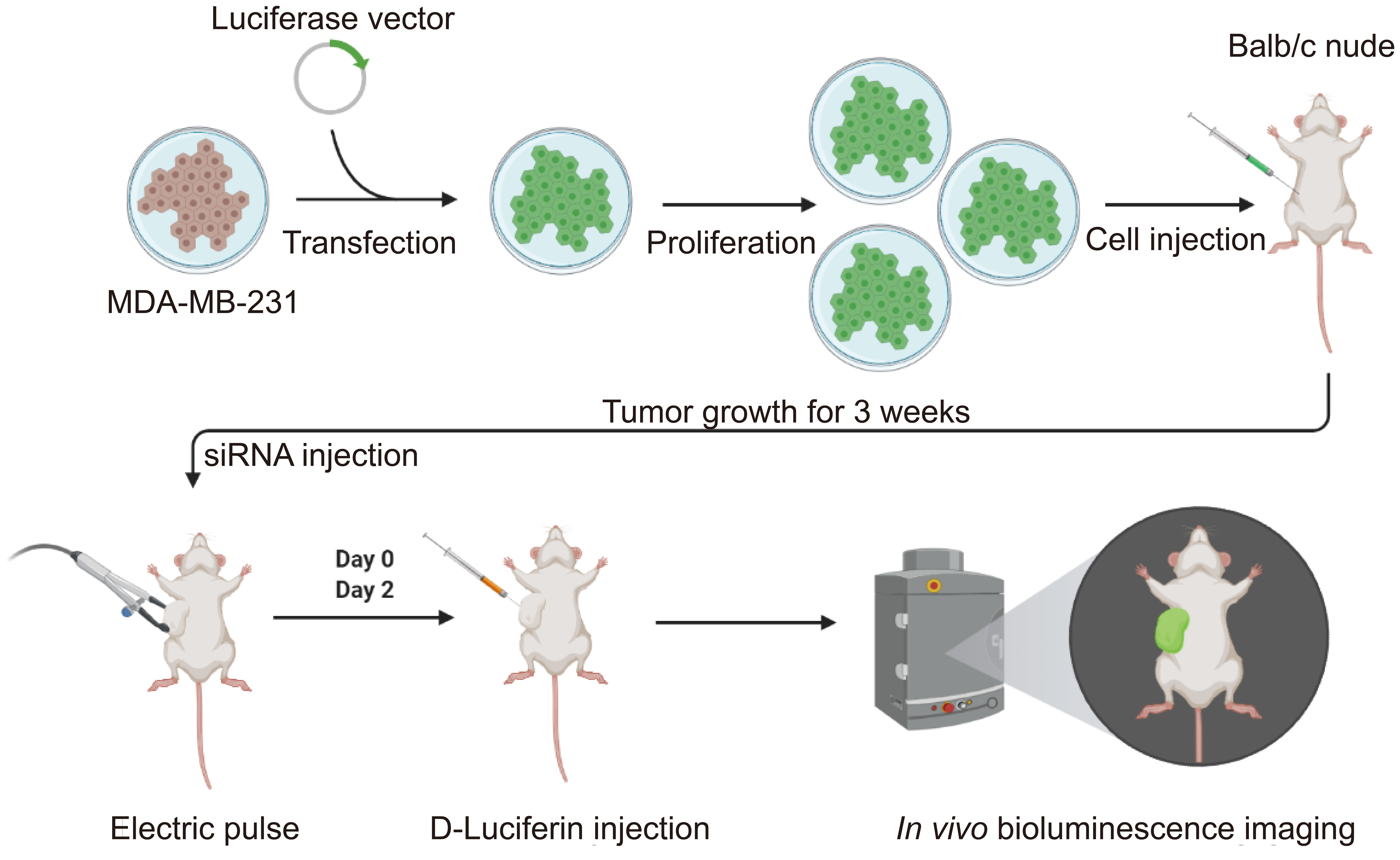


**Fig. S8 *In vivo* electroporation protocol.** Schematic cartoon of the xenograft mouse model construction process and electrotransfer procedure of siRNA delivery by means of a standard tweezer-type electrode. The bioluminescence intensity in this model was positively correlated with the expression level of luciferase stably expressed by MDA-MB-231-Luci tumor cells, allowing the bioluminescence levels to serve as indicators of gene expression.

| **Inoculation Time** | **Day 0** | | **Day 7** | | | |
| --- | --- | --- | --- | --- | --- | --- |
| **Block** | **Animal Number** | **Body Weight(g)** | **Body Weight(g)** | **L(mm)** | **W(mm)** | **V(mm³)** |
| **MDA-MB-231-Luci+30%Matrigel, 1×10^7^** | 1 | 22.00 | 22.20 | 6.03 | 5.37 | 86.94 |
|  | 2 | 20.80 | 20.70 | 6.28 | 4.87 | 74.47 |
|  | 3 | 21.70 | 22.10 | 6.99 | 4.91 | 84.26 |
|  | 4 | 21.80 | 21.70 | 6.82 | 5.26 | 94.35 |
|  | 5 | 21.40 | 21.30 | 6.83 | 4.80 | 78.68 |
|  | 6 | 21.10 | 22.40 | 5.50 | 4.87 | 65.22 |
|  | 7 | 21.40 | 22.00 | 6.01 | 4.54 | 61.94 |
|  | 8 | 18.20 | 20.20 | 7.37 | 5.69 | 119.31 |
|  | 9 | 22.20 | 22.60 | 7.64 | 5.39 | 110.98 |
|  | 10 | 22.50 | 21.80 | 7.65 | 5.00 | 95.63 |
|  | 11 | 21.00 | 20.80 | 6.47 | 5.25 | 89.16 |
|  | 12 | 21.30 | 21.30 | 6.35 | 5.09 | 82.26 |
|  | 13 | 22.80 | 23.50 | 8.03 | 5.61 | 126.36 |
|  | 14 | 21.30 | 21.60 | 7.14 | 5.24 | 98.02 |
|  | 15 | 20.90 | 21.50 | 8.29 | 5.46 | 123.57 |
|  | 16 | 21.40 | 22.00 | 6.69 | 4.40 | 64.76 |
|  | Mean | 21.36 | 21.73 | 6.88 | 5.11 | 90.99 |
|  | S.E | 0.25 | 0.20 | 0.19 | 0.09 | 5.15 |

**Table S3 Tumor volume after 7-day growth**


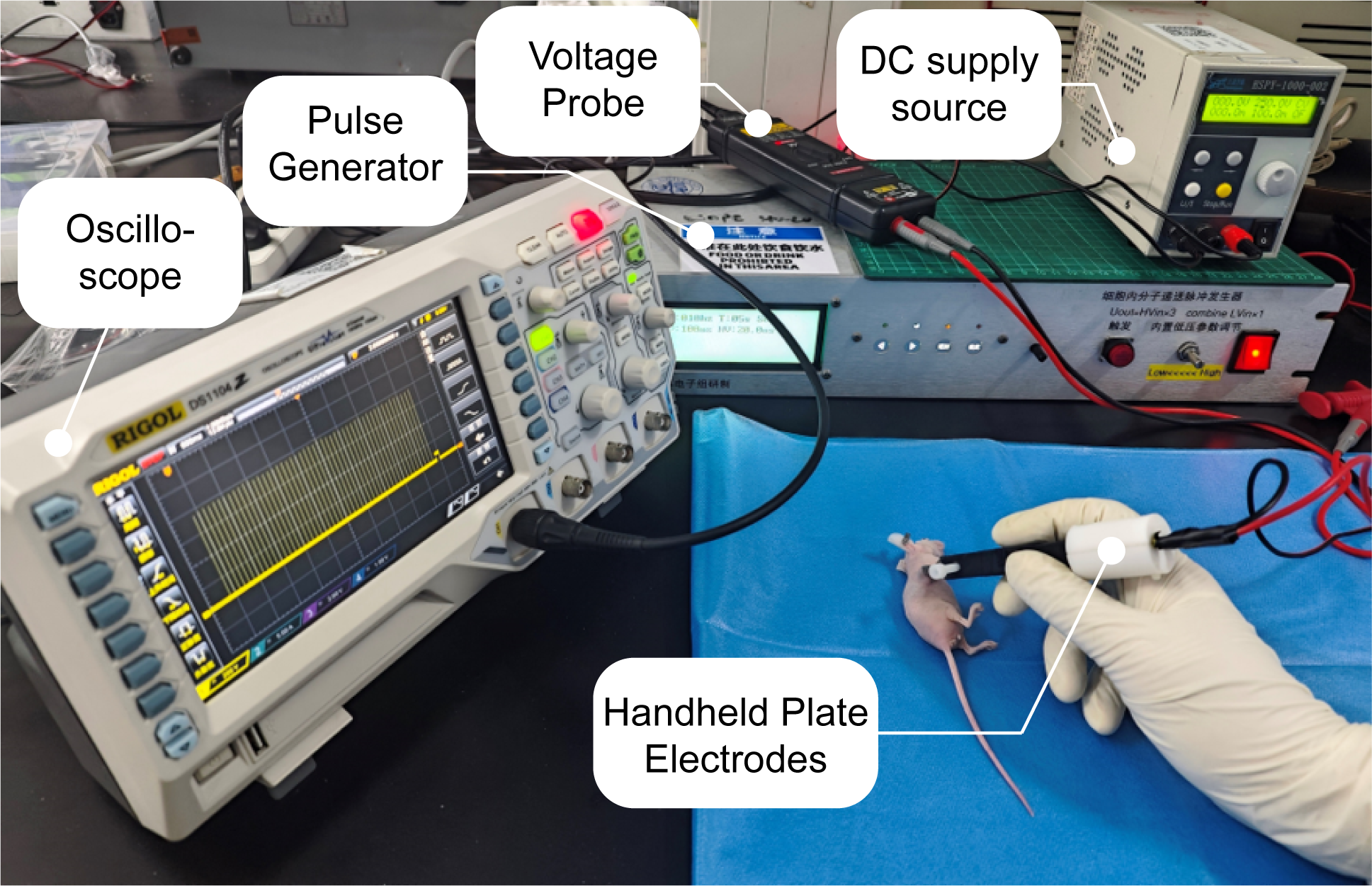


**Fig. S9 *In vivo* electroporation experimental device setup**


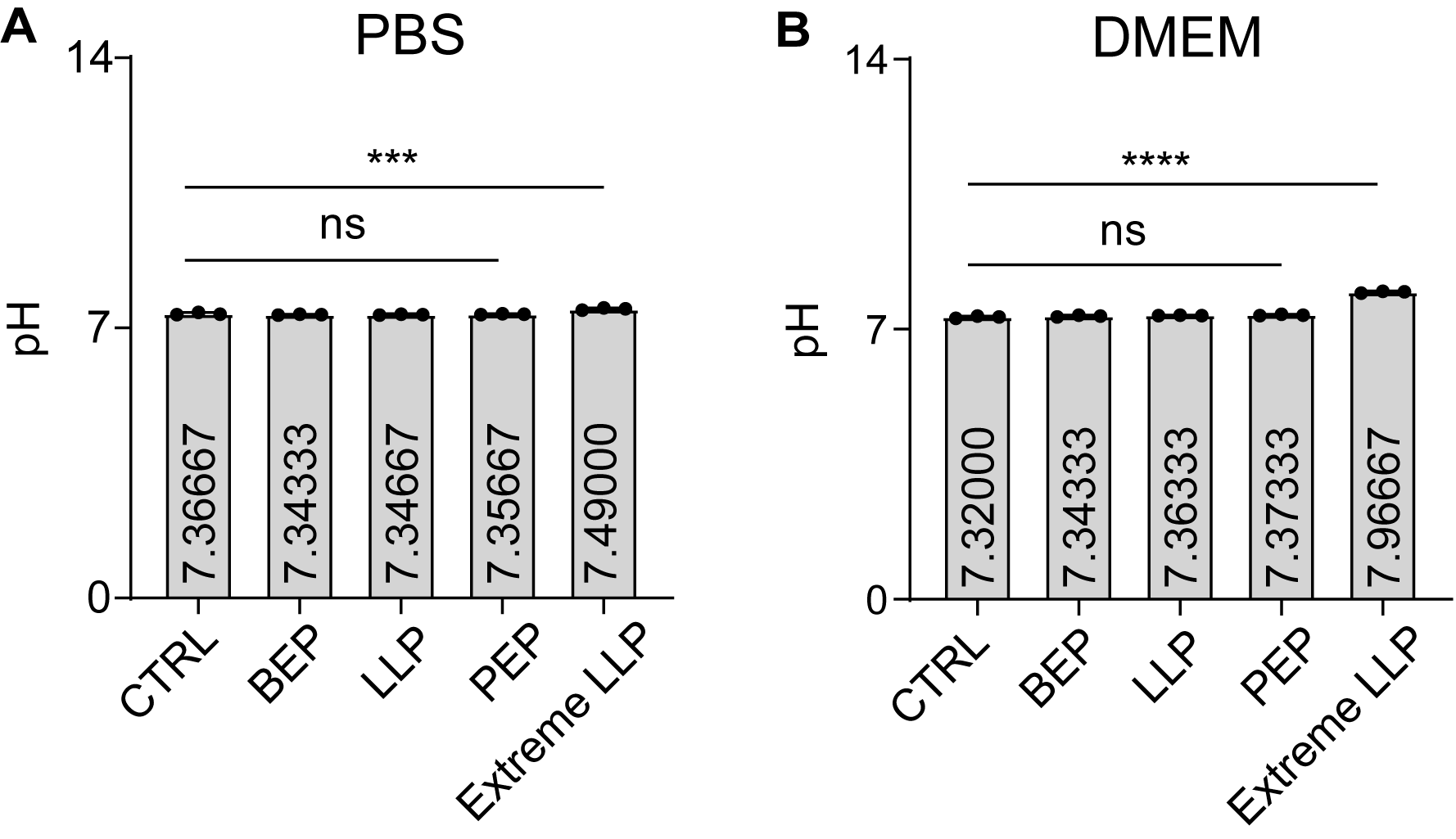


**Fig. S10 Changes in pH for electroporated buffer under different treatment.** (A) PBS. (B) DMEM. Results are expressed as mean ± standard error of the mean with 95% CI. Significance was evaluated by ordinary one-way ANOVA for multiple comparisons, ^***^p<0.001, ^****^p < 0.0001.

**
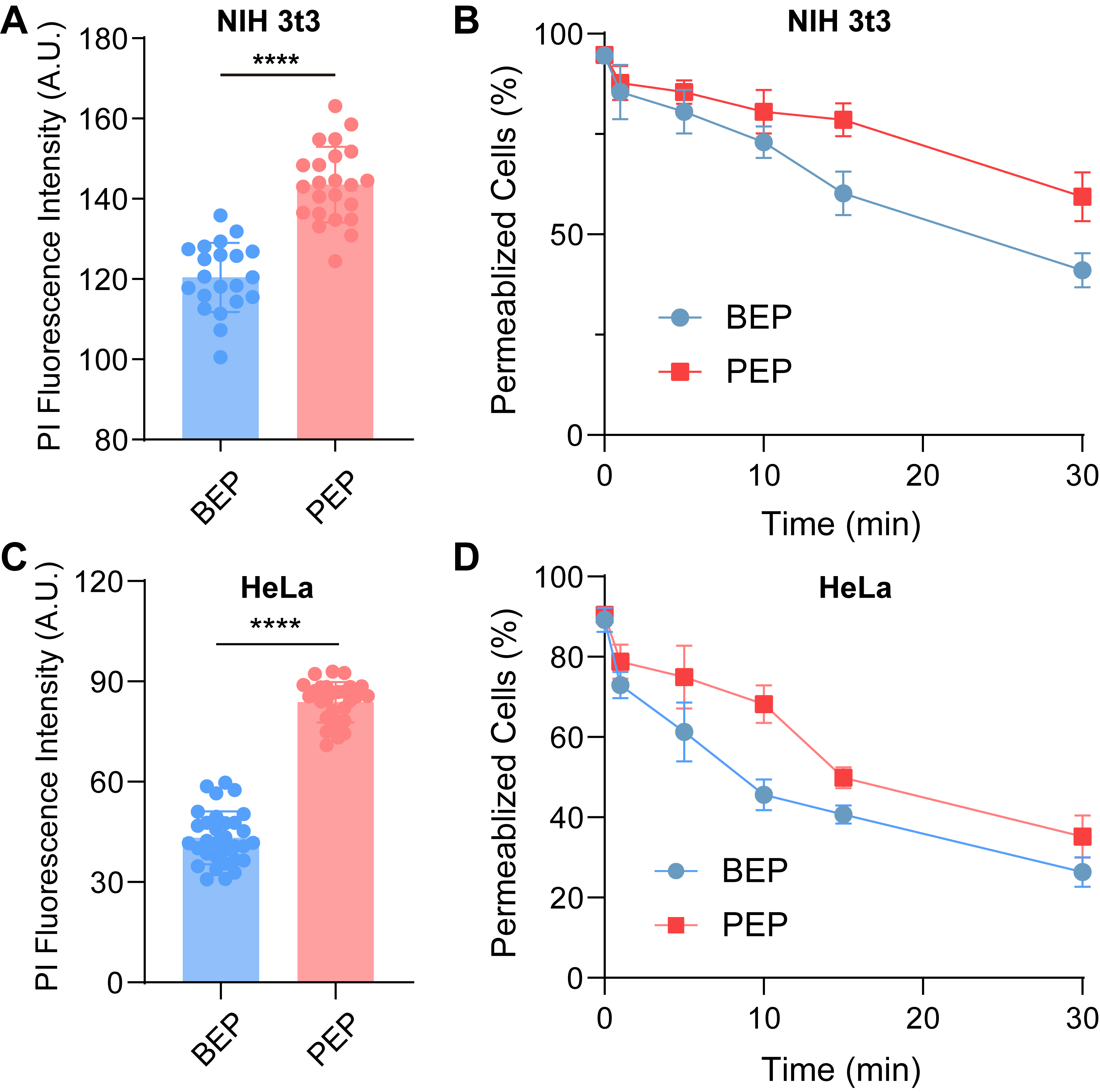
**

**Fig. S11 Regulation of pore resealing dynamics by progressive electroporation facilitated intracellular delivery.** (A) and (C), quantification of the fluorescence intensity. (B) and (D), dynamics of cell membrane resealing process electroporated by BEP and PEP (n = 4). (A) and (B), NIH-3t3 cells. (C) and (D), HeLa cells. Results are expressed as mean ± standard error of the mean with 95% CI. In (A) and (C), significance was evaluated by unpaired t-test for individual BEP and PEP groups, ****p < 0.0001.


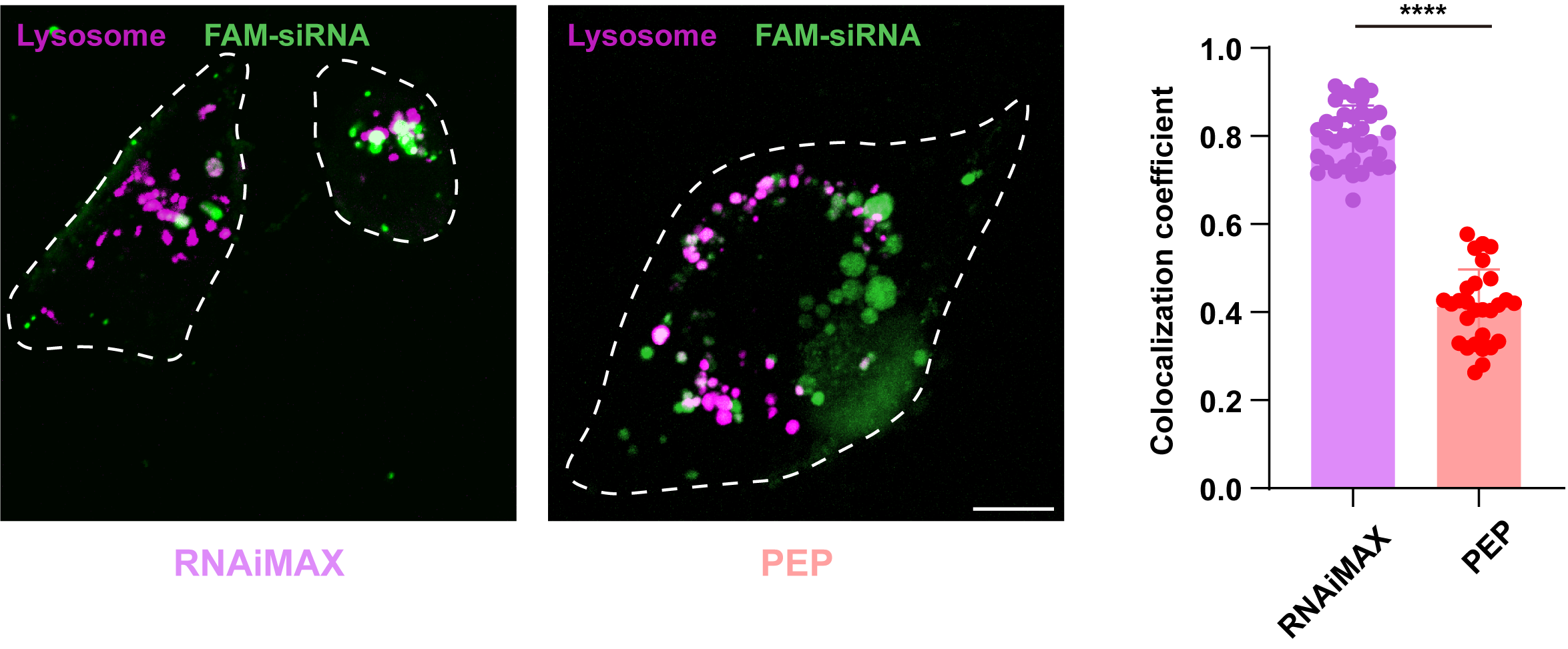


**Fig. S12 Colocalization analysis of lysosome and FAM-siRNA after two-hour incubation with RNAiMAX reagent or post PEP exposure.** Scale bar, 10 μm. Results are expressed as mean ± standard error of the mean with a 95% CI. Significance was evaluated by unpaired t-test for individual BEP and PEP groups, ****p < 0.0001.

**Method**

**NUMERICAL SIMULATION**

Here, we give comprehensive equations as the supplement for the main manuscript which govern processes between mesh nodes, along with parameters and their values. To evaluate the individual effect of two pulse components in progressive electroporation, a single high-voltage short-duration pulse (750 V/cm, 5 μs) and a single low-voltage long-duration pulse (75 V/cm, 200 μs) with no interval time were applied in the simulation.

***Electric Field Distribution***

A current source, $I_{rest}$, generates the resting membrane potential ^[3]^，

$\Delta\phi_{m,\text{ rest }}=I_{rest}R_{m},$ (1)

where $I_{rest}$ is established metabolically. The creation of pores reduces membrane resistance $R_{m}$, such that the membrane is typically depolarized post pulse. Electric field distribution can be calculated by solving Laplace equation and the development of transmembrane potential $\phi_{m}$ can be obtained by the Schwan equation for spherical model ^[10]^, which is in the form of (2),

$\Delta\phi_{m}=-1.5REcos\theta\left( 1-e^{\frac{-t}{\tau_{m}}} \right)$, (2)

where $\tau_{m}$ is the membrane charging constant,$R$ is the radius of cell,$E$ is the electric field intensity,$\theta$ is the polarization angle which refers to the angle between the direction opposite to the electric field. Schwan equation provides an insight into the spatial distribution and time dependence of transmembrane potential before the occurrence of membrane electroporation, which also indicates the critical transmembrane voltage for the breakdown of the membrane.

***Pore Energy***

Pore creation and destruction are considered stochastic on the membrane. As mentioned previously, the fluctuation of the pore was dominantly command by pore energy. $W$ is derived from four contributions ^[9, 11]^,

$W\left( r_{p},V_{m\text{ }} \right)=W_{steric}\left( r_{p} \right)+W_{edge}\left( r_{p} \right)+W_{surf}\left( r_{p} \right)+W_{elec}\left( r_{p},V_{m\text{ }} \right)$, (3)

Steric repulsion of the lipid head groups gives as follows,

$W_{steric}\left( r_{p} \right)=\beta\left( \frac{r^{*}}{r_{p}} \right)^{4}+C,$ (4)

where$\beta$ and $C$ are constants.

Edge energy accounts for bending of the lipid around the circumference of a pore,

$W_{edge}\left( r_{p} \right)=2\pi\gamma r_{p}$, (5)

where $\gamma$ is the line tension. The interfacial energy is in the form of (6),

$W_{edge}\left( r_{p} \right)=-\pi\delta{\cdot\Delta A}_{p}\cdot r_{p}^{2},$ (6)

where $\delta={10}^{-5}N/m$ is the initial membrane tension varying with electroporation process and ${\Delta A}_{p}$ is the decrease in membrane area due to a pore. The electrical energy contribution, $W_{elec}\left( r_{p},\Delta\phi_{m} \right)$ is

$W_{elec}=-\int_{0}^{r_{p}} F\left( r \right)dr=-F_{max}\left( r_{p}+r_{h}\ln\left( \frac{r_{t}+r_{h}}{r_{p}+r_{t}+r_{h}} \right) \right)\left( \Delta\phi_{m} \right)^{2}$, (7)

where $F_{\max}$ is the maximum pore expanding force, and $r_{h}$ and $r_{t}$ are constants.

***Pore Dynamics***

An electroporation response involves pore creation, expansion, contraction, and destruction. The pore flux $J_{p}$ per unit area in pore radius space is ^[8]^

$J_{p}=-D_{p}\frac{\partial n}{\partial r_{p}}-\frac{D_{p}}{kT}n\frac{\partial W}{\partial r_{p}}$, (8)

where $n$ is the pore density per unit area per $dr_{p}$ and $D_{p}$ is the pore diffusion coefficient in pore radius space. $k$ is the Boltzmann constant and $T$ is the temperature. When external energy is injected, the second term in the equation plays a leading role, and the pore evolution equation can be simplified as follows ^[10, 12]^,

$\frac{{dr}_{p}}{dt}=-\frac{D_{p}}{kT}\frac{\partial W}{\partial r_{p}}$. (9)

The evolution of pores on the cell membrane during electroporation is thought to be related to the synergistic effect of the surface tension of the cell membrane, repulsive force of lipid heads, and edge line tension on the pore perimeter, composing the energy landscape $W_{total}$ of the pore, which determines the evolution of the pore radius. Time-dependent changes of the pore radius can be quantified by differential equations ^[11]^,

$$\frac{{dr}_{p,i}}{dt}=U\left（ r_{p,i},\phi_{m}, \delta_{eff} \right）={(D}/{kT})(\frac{{\phi_{m}}^{2}F_{max}}{1+{r_{h}}/\left( r_{p,i}+r_{t} \right)}+ 4\beta\left( \frac{r^{*}}{r_{p,i}} \right)^{4} \frac{1}{r_{p,i}}-2\pi\gamma+ 2\pi\delta_{eff}r_{p,i})$$

$i=1, 2, \ldots, K$ (10)

where *K* is the total number of pores,$D$ is the diffusion coefficient of the pore interior,$F_{max}$ is the maximum electric force, and $r_{t}$and $r_{h}$ are constants for the advection velocity. $\beta$ is the steric repulsion energy, $r^{*}$ is the minimum radius of the hydrophilic pores, and $\gamma$ is the edge energy. $\delta_{eff}$ is the effective tension coefficient of the cell membrane and is defined by ^[8]^

$\delta_{eff}=2\sigma^{'}-\left( 2\sigma^{'}-\sigma_{0} \right)/{{(1-{A_{p}}/{A_{s})}}^{2}}$, (11)

where $\sigma_{0}$ and $\sigma^{'}$ are the tension of the intact cell membrane and energy of the hydrocarbon-water interface per area, respectively,$A_{p}$ is the total area of the pores and can be described by $\sum_{i=1}^{K} \pi{r_{p,i}}^{2}$, and $A_{s}$ is the total area of the cell membrane. To simplify the equation of the pore radius, we assumed the population of the pores was homogeneous and redefined $A_{p}=A_{s}\cdot N\pi{r_{p,i}}^{2}$ in this model.$N$ is the pore density, which can be obtained by simplified Smoluchowski equation ^[13]^,

$\frac{dN}{dt}=\alpha e^{\left( \frac{\phi_{m}}{V_{ep}} \right)^{2}}\left( 1-\frac{N}{N_{0}}e^{-q\left( \frac{\phi_{m}}{V_{ep}} \right)^{2}} \right)$, (12)

where $\alpha$ is a coefficient constant, $V_{ep}$is the characteristic voltage of electroporation, and $N_{0}$is the pore density when the transmembrane potential is zero. The flow of contracting pores from larger radii to $r^{*}$ occurs predominantly after the pulse, when $V_{m\text{ }}=0$. The pore energy landscape governs the energetics of drift and diffusion in the pore radius space, such that the pore lifetime $\tau_{p}$ is approximately related to the destructive pore energy barrier $W_{d}$ by ^[14-15]^,

$\tau_{p}\approx\frac{\left( r_{min}-r^{*} \right)^{2}}{D_{p}}\left( \frac{W_{d}}{kT} \right)^{-\frac{1}{2}}e^{\frac{W_{d}}{kT}}$, (13)

$W_{d}=W\left( r^{*} \right)-W(r_{min})$, (14)

***Solute Electrodiffusion in Bulk Media***

The migration of molecules in the cell suspension follows the Nernst-Planck equation. The electrodiffusive flux $J_{s}$ in a bulk electrolyte solution in 1-D condition ($K=1;$ no partitioning or hindrance) is ^[16]^,

$J_{c}=-\left( D_{c, dif}\frac{\partial n}{\partial x}+\frac{D_{c, dif}q_{e}z_{c}}{kT}n_{c}\frac{\partial\varphi}{\partial x} \right)$, (15)

where $J_{c}$ is the flux of the solute, $n_{c}$ is the molecule concentration, $D_{c, dif}$ is the diffusion coefficient of the solute, $z_{c}$ is the effective valence, $\varphi$ is the electric potential, $q_{e}$ is the elementary charge, and $x$ is the unit length. According to ^[17-18]^, the diffusion coefficient of particles through the cell membrane can be expressed as follows，

$D_{m}=0.01\times D_{c, dif}\left( 1-p \right)+D_{c, dif}p$, (16)

where$p$=$N\pi{r_{p, i}}^{2}$, indicating the permeability of membrane. Because charged particles have difficulty entering the cell in a static state, the cell membrane thickness can be considered as a unit length. If the membrane is seen as a diffusion barrier, by 1-D Nernst-Planck equation, the charged particle flux across the membrane can be written as follows ^[2]^,

$\frac{\partial n}{\partial t}=-\nabla\cdot(\frac{J_{m,c}}{Fz_{c}})$, (17)

$J_{m,c}={-Fz_{c}\cdot D}_{m}\left[ \frac{\partial n}{\partial x}+\frac{q_{e}z_{c}}{k_{B}T}c\left( \frac{\partial\varphi}{\partial x} \right) \right]$, (18)

***Partition Factor***

The equilibrium concentration of a charged solute is smaller inside a pore than in the bulk solution. This decrease results from the energy cost of moving a charge from the bulk aqueous medium (high dielectric constant) into the pore interior (low dielectric constant lipid). The partition factor $K$ plays a role in determining the transport of a charged solute through pores and is defined as ^[14]^,

$K=K\left( r_{p},\Delta\psi_{p} \right)= \frac{e^{\Delta\psi_{p}}-1}{\frac{\omega_{0}e^{\omega_{0}-\eta\Delta\psi_{p}}-\eta\Delta\psi_{p}}{\omega_{0}-\eta\Delta\psi_{p}}e^{\Delta\psi_{p}}-\frac{\omega_{0}e^{\omega_{0}+\eta\Delta\psi_{p}}+\eta\Delta\psi_{p}}{\omega_{0}+\eta\Delta\psi_{p}}}$, (19)

where the dimensionless electrostatic transpore voltage $\Delta\psi_{p}$ is defined as ^[16]^

$\Delta\psi_{p}\equiv\frac{q_{e}z_{eff}}{kT}\Delta\phi_{p}$, (20)

where $\Delta\phi_{p}$is the transpore voltage. The entrance region length $\eta$ of a trapezoidal pore is $d_{mem}/4$, and $\omega_{0}$ is the Born energy. According to the two-dimensional Cartesian network model, the pore resistance is in parallel with the resistors of the cell membrane, and hence the transpore voltage $\Delta\phi_{p}$ can be defined by the transmembrane voltage $\Delta\phi_{m}$ as follows,

$\Delta\phi_{p}=\Delta\phi_{m}$. (21)

The particles are uniformly distributed in the pore and the particle transport flux is continuous. The total membrane conductivity is ^[2]^

$\sigma_{mem}=\sigma_{m0}+\sigma_{pore}(t)=\sigma_{m0}+KN\pi r_{p}^{2}\sigma_{p}$, (22)

where $\sigma_{m0}$ is initial membrane conductivity and $\sigma_{p}$ is the pore conductivity.

***Solute Electrodiffusion Discretized Through a Pore of Radius*** $\boldsymbol{r}_{\boldsymbol{p}}$

For each pore in the distribution of pore radii, the solute transport is estimated using

$J_{s,p}=K\left( r_{p} \right)J_{s}$, (23)

where $K$ is the partition factor. The solute current through a pore is ^[16]^

$i_{s,p}=\pi r_{p}^{2}K\left( r_{p} \right)J_{s}$, (24)

and the transmembrane flux is ^[14, 16]^

$J_{s,m}=N\pi r_{p}^{2}K\left( r_{p} \right)J_{s}$, (25)

These equations are solved between the nodes of the 2-D mesh of the system model and at each solver time step, with time-varying pore distributions and transmembrane voltages at each membrane node pair. Kirchhoff's laws are used to connect the transmembrane solute fluxes to the portion of the meshed network residing within the extra-and intracellular aqueous media ^[2]^.

**Reference**

[1] E. Goldberg, C. Suárez, M. Alfonso, J. Marchese, A. Soba, G. Marshall, *Bioelectrochemistry* **2018**, 124, 28.

[2] H. Liu, X. Tao, X. Xiang, H. Zhao, J. Qiu, K. Liu, *IEEE Transactions on Dielectrics and Electrical Insulation* **2023**, DOI: 10.1109/TDEI.2023.33272181.

[3] K. A. DeBruin, W. Krassowska, *Biophysical Journal* **1999**, 77, 1213.

[4] A. O. Bilska, K. A. DeBruin, W. Krassowska, *Bioelectrochemistry* **2000**, 51, 133.

[5] M. A. Chiapperino, L. Mescia, P. Bia, B. Starešinič, M. Čemažar, V. Novickij, A. Tabašnikov, S. Smith, J. Dermol-Černe, D. Miklavčič, *IEEE Transactions on Biomedical Engineering* **2020**, 67, 2781.

[6] O. Ma, M. Zhang, *IEEE Transactions on Automation Science and Engineering* **2009**, 6, 228.

[7] K. A. DeBruin, W. Krassowska, *Annals of Biomedical Engineering* **1998**, 26, 584.

[8] W. Krassowska, P. D. Filev, *Biophysical Journal* **2007**, 92, 404.

[9] J. C. Neu, W. Krassowska, *Physical Review E* **1999**, 59, 3471.

[10] T. Kotnik, L. Rems, M. Tarek, D. Miklavčič, *Annual Review of Biophysics* **2019**, 48, 63.

[11] J. C. Neu, K. C. Smith, W. Krassowska, *Bioelectrochemistry* **2003**, 60, 107.

[12] M. Scuderi, J. Dermol-Černe, C. Amaral da Silva, A. Muralidharan, P. E. Boukany, L. Rems, *Bioelectrochemistry* **2022**, 147, 108216.

[13] A. Barnett, J. C. Weaver, *Journal of Electroanalytical Chemistry and Interfacial Electrochemistry* **1991**, 320, 163.

[14] K. Smith, **2011**.

[15] V. F. Pastushenko, Y. A. Chizmadzhev, V. B. Arakelyan, *Journal of Electroanalytical Chemistry and Interfacial Electrochemistry* **1979**, 104, 53.

[16] K. C. Smith, J. C. Weaver, *IEEE Transactions on Biomedical Engineering* **2012**, 59, 1514.

[17] F. Guo, K. Qian, L. Zhang, X. Liu, H. Peng, *Bioelectrochemistry* **2021**, 141, 107878.

[18] A. Barnett, *Biochimica et Biophysica Acta (BBA) - Biomembranes* **1990**, 1025, 10.
